# Structural Basis of the Heterodimer Formation between Cell Shape-Determining Proteins Csd1 and Csd2 from *Helicobacter pylori*

**DOI:** 10.1371/journal.pone.0164243

**Published:** 2016-10-06

**Authors:** Doo Ri An, Ha Na Im, Jun Young Jang, Hyoun Sook Kim, Jieun Kim, Hye Jin Yoon, Dusan Hesek, Mijoon Lee, Shahriar Mobashery, Soon-Jong Kim, Se Won Suh

**Affiliations:** 1 Department of Biophysics and Chemical Biology, College of Natural Sciences, Seoul National University, Seoul, Korea; 2 Department of Chemistry, College of Natural Sciences, Seoul National University, Seoul, Korea; 3 Biomolecular Function Research Branch, Division of Precision Medicine and Cancer Informatics, Research Institute, National Cancer Center, Gyeonggi, Korea; 4 Department of Chemistry and Biochemistry, University of Notre Dame, Notre Dame, Indiana, United States of America; 5 Department of Chemistry, Mokpo National University, Chonnam, Korea; Russian Academy of Medical Sciences, RUSSIAN FEDERATION

## Abstract

Colonization of the human gastric mucosa by *Helicobacter pylori* requires its high motility, which depends on the helical cell shape. In *H*. *pylori*, several genes (*csd1*, *csd2*, *csd3*/*hdpA*, *ccmA*, *csd4*, *csd5*, and *csd6*) play key roles in determining the cell shape by alteration of cross-linking or by trimming of peptidoglycan stem peptides. *H*. *pylori* Csd1, Csd2, and Csd3/HdpA are M23B metallopeptidase family members and may act as d,d-endopeptidases to cleave the d-Ala^4^-*m*DAP^3^ peptide bond of cross-linked dimer muropeptides. Csd3 functions also as the d,d-carboxypeptidase to cleave the d-Ala^4^-d-Ala^5^ bond of the muramyl pentapeptide. To provide a basis for understanding molecular functions of Csd1 and Csd2, we have carried out their structural characterizations. We have discovered that (i) Csd2 exists in monomer-dimer equilibrium and (ii) Csd1 and Csd2 form a heterodimer. We have determined crystal structures of the Csd2_121–308_ homodimer and the heterodimer between Csd1_125–312_ and Csd2_121–308_. Overall structures of Csd1_125–312_ and Csd2_121–308_ monomers are similar to each other, consisting of a helical domain and a LytM domain. The helical domains of both Csd1 and Csd2 play a key role in the formation of homodimers or heterodimers. The Csd1 LytM domain contains a catalytic site with a Zn^2+^ ion, which is coordinated by three conserved ligands and two water molecules, whereas the Csd2 LytM domain has incomplete metal ligands and no metal ion is bound. Structural knowledge of these proteins sheds light on the events that regulate the cell wall in *H*. *pylori*.

## Introduction

*Helicobacter pylori* is a Gram-negative bacterium that colonizes the stomach of roughly half of the world’s population, thus causing a variety of gastrointestinal diseases such as peptic ulcer and gastric cancer [1]. It is regarded as a primary factor for gastric cancer development [1] and is the sole Group I carcinogen among pathogenic bacteria, according to the classification by the International Agency for Research on Cancer. Recent reports also suggest possible links between *H*. *pylori* infection and some extra-digestive diseases, including neurodegenerative disorders [2]. Typical treatment regimens for *H*. *pylori* infection consist of a proton pump inhibitor such as omeprazole and the antibiotics clarithromycin and amoxicillin (or metronidazole). However, increasing drug resistance requires new therapies and the discovery of new antibiotics [3].

High motility of *H*. *pylori* is important for its colonization of the human stomach and its survival in its preferred niche, the gastric mucosa [4–6]. The helical cell shape of *H*. *pylori* is believed to facilitate efficient colonization of the viscous epithelial mucus layer via a cork-screwing mechanism [7–9]. Mutants of *H*. *pylori* with altered cell shapes exhibited attenuated colonization [10,11]. The peptidoglycan layer of a bacterial cell wall plays a key role not only in protecting cells against the intracellular turgor pressure but also in maintaining the proper cell shape [12–14]. It is made of linear polysaccharide chains that consist of alternating β-1,4-linked *N*-acetylglucosamine-*N*-acetylmuramic acid (NAG-NAM) disaccharide units, with a pentapeptide covalently linked to NAM [15]. The pentapeptide chains are either 4→3 or 3→3 cross-linked to different extents depending on bacterial species. In *H*. *pylori*, the pentapeptide consists of l-Ala^1^-*γ*-d-Glu^2^-*m*DAP^3^-d-Ala^4^-d-Ala^5^ (or -Gly^5^), where *m*DAP refers to *meso*-2,6-diaminopimelate. The neighboring peptides are cross-linked exclusively by the 4→3 linkage between the α-carboxylic group of d-Ala^4^ on one strand (the donor peptide) and the ε-amino group of *m*DAP^3^ on another strand (the acceptor peptide) [16,17] to form a mesh-like peptidoglycan (murein) sacculus [18]. In many bacteria, the peptidoglycan layer is remodeled by a number of peptidoglycan hydrolases and by lytic transglycosylases that function in the peptidoglycan maturation, regulation of cell wall growth, cell division, peptidoglycan turnover and recycling, cell lysis and the release of peptidoglycan fragments for host–pathogen interactions [19–23].

In *H*. *pylori*, cleaving the 4→3 cross-links of peptidoglycan or trimming of peptidoglycan muropeptides affect its helical cell shape. A small number of proteins have been identified to be essential in generating the helical cell shape of *H*. *pylori* by tailoring the peptidoglycan layer [11]: an amidase AmiA [24], potential peptidoglycan peptidases Csd1–Csd4 [10,25,26] and Csd6 [27], and potential regulators Csd5 and CcmA [10,25]. We have recently reported the crystal structures of Csd6 (HP0518 in *H*. *pylori* 26695 strain) [28], Csd4 (HP1075) [29], and Csd3 (HP0506) [30]. We have shown that *H*. *pylori* Csd6 is dimeric, with each monomer consisting of three domains: an N-terminal domain, a middle l,d-carboxypeptidase domain, and a C-terminal NTF2-like domain. We have also shown that *H*. *pylori* Csd6 constitutes a new family of l,d-carboxypeptidase, with the active-site in its ‘l,d-transpeptidase’ domain being tailored to function as l,d-carboxypeptidase to convert the muramyl tetrapeptide into the muramyl tripeptide by cleaving the *m*DAP^3^-d-Ala^4^ bond; Csd6 is nonfunctional as an l,d-transpeptidase [28]. *H*. *pylori* Csd4 is a Zn^2+^-dependent d,l-carboxypeptidase of the M14 metallopeptidase family and cleaves the γ-d-Glu^2^-*m*DAP^3^ bond of the uncross-linked muramyltripeptide (muramyl-l-Ala^1^-γ-d-Glu^2^-*m*DAP^3^) of the peptidoglycan to produce the muramyldipeptide (muramyl-l-Ala^1^-γ-d-Glu^2^) and *m*DAP [25,29]. *H*. *pylori* Csd4 is monomeric, with a monomer consisting of three domains: an N-terminal carboxypeptidase domain, a central β-barrel domain of a novel fold, and a C-terminal immunoglobulin-like domain [29,31]. *H*. *pylori* Csd3 (also known as HdpA) belongs to the M23B metallopeptidase family and possesses not only the d,d-endopeptidase activity to cleave the 4→3 cross-link but also the d,d-carboxypeptidase activity that cleaves the d-Ala^4^-d-Ala^5^ bond of the muramyl pentapeptide to produce the muramyl tetrapeptide [10,11,26]. We have recently reported the crystal structure of the N-terminally truncated Csd3 encompassing residues 42–403 [30]. It is monomeric and consists of three domains: Domain 1 (residues 42–124), Domain 2 (residues 125–228 and 360–403) and the C-terminal LytM domain (residues 229–359). The LytM domain of Csd3 has the canonical fold with a zinc-containing active site and Domain 1 functions as an inhibitory domain by blocking the access of the substrate into the active site in the latent state [30].

Two Csd proteins, Csd1 (HPG27_1481 in *H*. *pylori* G27 strain, HP1543 in *H*. *pylori* 26695 strain) and Csd2 (HPG27_1482 in *H*. *pylori* G27 strain, HP1544 in *H*. *pylori* 26695 strain), were identified to contain the LytM domain [10]. *H*. *pylori* Csd1 and Csd2 belong to the M23B metallopeptidase family and may act as the d,d-endopeptidase to cleave the 4→3 cross-links [10,11]. Csd1 shows the broadest conservation with a homolog present in most ɛ-proteobacteria, whereas Csd2 homologs are found only in *H*. *pylori* and *H*. *hepaticus* [10]. Csd3 homologs, which contain an N-terminal extension not present in Csd1 and Csd2, are well conserved throughout the ɛ-proteobacteria including *H*. *pylori* [10]. All the Csd1 and Csd3 homologs identified preserve conserved residues predicted to function in catalysis as peptidoglycan endopeptidases or carboxypeptidases [10]. To provide insight into the molecular functions of *H*. *pylori* Csd1 and Csd2 proteins, we have carried out their structural characterizations. We have discovered that (i) Csd2 exists in a monomer-dimer equilibrium in solution by equilibrium sedimentation and (ii) Csd1 and Csd2 form a stable 1:1 heterodimer. We have determined the crystal structures of the Csd2_121–308_ homodimer and the heterodimer between Csd1_125–312_ and Csd2_121–308_. The overall structures of Csd1_125–312_ and Csd2_121–308_ monomers are similar to each other, consisting of a helical domain and a LytM domain. The helical domains of both Csd1 and Csd2 play a key role in the formation of homodimers or heterodimers. LytM domains of Csd1 and Csd2 share the same overall fold but a significant difference exists in their active sites. The Csd1 LytM domain contains a catalytic site with a Zn^2+^ ion, which is coordinated by three conserved ligands and two water molecules, whereas Csd2 has a degenerate LytM domain with incomplete metal ligands and no metal ion is bound. We have also observed two types of non-canonical Zn^2+^-coordination in the active site of Csd1 LytM domain. In one Csd2_121–308_ chain of the heterodimer models between Csd1_125–312_ and Csd2_121–308_, the C-terminal tail of Csd2 is bound to the central groove of the Csd1 LytM domain and defines the substrate binding site. The structural knowledge from this work could serve as the foundation in discovery of novel inhibitors that would prove helpful in fighting infections by the major human pathogen *H*. *pylori*.

## Materials and Methods

### Expression and purification of Csd2

Five different constructs of Csd2 (residues 54–308, 63–308, 77–308, 121–308, and140–251) were individually expressed in a soluble form. Among them, crystals were produced from the Csd2_121–308_ construct only. Expression and protein purification of Csd2_121–308_ are given as a representative example below.

For overexpression of Csd2_121–308_, the *csd2* gene (HP1544 from *H*. *pylori* 26695 strain) was PCR-amplified and was cloned into the expression vector pET-28b(+) (Novagen). The resulting recombinant Csd2_121–308_ is fused with hexahistidine-containing tags at both N- and C-termini (MGSSHHHHHHSSGLVPRGSH at the N-terminus and LEHHHHHH at the C-terminus). To perform SEC-MALS (size-exclusion chromatography with multi-angle static light scattering) and equilibrium sedimentation experiments, we also PCR-amplified the *csd2* gene covering residues 140–251 and cloned it into the expression vector pET-21a(+) (Novagen) to express the recombinant Csd2_140–251_protein fused with a hexahistidine-containing tag (LEHHHHHH) at the C-terminus. We followed identical procedures for cell culture and protein purification of both Csd2_121–308_ and Csd2_140–251_. The recombinant proteins were overexpressed in *Escherichia coli* Rosetta 2(DE3)pLysS cells, using the Luria Broth culture medium. Protein expression was induced by 0.5 mM isopropyl β-d-thiogalactopyranoside and the cells were incubated for additional 15 h at 30°C following growth to mid-log phase at 37°C. The cells were harvested and were suspended in an ice-cold lysis buffer [20 mM Tris-HCl at pH 7.9, 500 mM sodium chloride, 50 mM imidazole, and 10% (v/v) glycerol] containing 1 mM phenylmethylsulfonyl fluoride. The cells were lysed by sonication. After centrifugation at 36,000 *g* for 1 h at 4°C, the cell debris was discarded and the supernatant was applied to an affinity chromatography column of HiTrap Chelating HP (GE Healthcare), which was previously equilibrated with the lysis buffer. The column was washed with the lysis buffer containing 25 mM imidazole, and was eluted with a linear gradient from 25 to 500 mM imidazole. The recombinant Csd2_121–308_ and Csd2_140–251_ proteins eluted at 150–200 mM and 120–150 mM imidazole concentrations, respectively. The eluted protein was further purified by gel filtration on a HiLoad 16/60 Superdex 200 prep-grade column (GE Healthcare), which was previously equilibrated with 20 mM HEPES at pH 7.5 and 200 mM sodium chloride. Peak fractions containing the Csd2_121–308_ protein were pooled and concentrated to 10 mg/ml (0.20 mM homodimer concentration) for crystallization.

### Expression and purification of Csd1-Csd2 complexes

We initially tried to express and purify the Csd1 protein alone using four different constructs. The construct covering residues 125–312 of *H*. *pylori* Csd1 (HP1543 from 26695 strain) was cloned into the expression vector pET-28b(+), resulting in Csd1_125–312_ fused with hexahistidine-containing tags at both N- and C-termini. Three other constructs were also PCR-amplified and were cloned into the expression vector pET-21a(+), resulting the recombinant Csd1_54–312_ (residues 54–312), Csd1_75–312_ (residues 75–312), and Csd1_91–312_ (residues 91–312) proteins, which are fused with a hexahistidine-containing tag at the C-terminus. All recombinant Csd1 proteins were overexpressed in *E*. *coli* Rosetta 2(DE3)pLysS cells using the Luria Broth culture medium. Protein expression was induced by 0.5 mM isopropyl β-d-thiogalactopyranoside and the cells were incubated for additional 15 h at 30°C following growth to mid-log phase at 37°C. All of the above Csd1 constructs were expressed in an insoluble form despite extensive screening of the cell culture condition.

As explained above, we found that Csd2_121–308_ forms a homodimer in the crystal and exists in monomer-dimer equilibrium in solution. Csd2_140–251_ also exists in monomer-dimer equilibrium in solution. Therefore, we tested the possible complex formation between the above four Csd1 constructs and Csd2_121–308_ without a fusion tag. The Csd2_121–308_ construct was cloned into the expression vector pET-21a(+) to express the recombinant Csd2_121–308_ without a hexahistidine-containing tag at both N- or C-termini. Cells expressing each of the above four Csd1 constructs with fusion tag(s) and Csd2_121–308_ without a fusion tag were grown separately. Cells were mixed in an approximate mass ratio of 3:1 for Csd1 and Csd2 to account for different expression levels. The mixed cells were diluted in the ice-cold lysis buffer containing 1 mM phenylmethylsulfonyl fluoride and lysed using sonication. After centrifugation at 36,000 *g* for 1 h at 4°C to discard the cell debris, the supernatant was applied to the affinity chromatography column of HiTrap Chelating HP (GE Healthcare), which was previously equilibrated with the lysis buffer. The column was washed with the lysis buffer containing 25 mM imidazole, and eluted with a linear gradient from 25 to 500 mM imidazole. The complexes between each of the above four Csd1 constructs and Csd2_121–308_ were eluted at 150–250 mM imidazole concentration. The complex formation was confirmed by SDS-PAGE. We further confirmed by SEC-MALS that Csd1_54–312_ and Csd2_121–308_ form a heterodimer in solution. For crystallization, the complex between Csd1_125–312_ and Csd2_121–308_ was further purified by gel filtration on a HiLoad 16/60 Superdex 200 prep-grade column (GE Healthcare), which was previously equilibrated with 20 mM HEPES at pH 7.5 and 200 mM sodium chloride. Peak fractions containing the Csd1_125–312_ and Csd2_121–308_ complex were pooled and concentrated to 8 mg/ml (0.15 mM heterodimer concentration) for crystallization.

### Crystallization and X-ray data collection

Crystals of Csd2_121–308_ were grown at 23°C by the sitting-drop vapor diffusion method using the Mosquito robotic system (TTP Labtech). Each sitting drop (0.4 μl) was prepared by mixing equal volumes of the protein solution at 10 mg/ml and the reservoir solution [100 mM HEPES at pH 7.0, and 30% (w/v) Jeffamine ED-2001]. The sitting drop was equilibrated against 100 μl of the reservoir solution. Rod-shaped crystals grew up to approximate dimensions 0.3 mm × 0.05 mm × 0.05 mm in 3 days. They were cryoprotected in the reservoir solution supplemented with 10% (v/v) glycerol, and were flash-frozen in a nitrogen gas stream at 100 K. Native data were collected to 1.80 Å resolution using the ADSC Q315r CCD detector at the beamline BL-5C of Pohang Light Source, Pohang, Korea. Raw X-ray diffraction data were processed and scaled using the program suit HKL2000 [32]. Assuming the presence of one Csd2_121–308_ chain in the asymmetric unit, the Matthew’s coefficient and solvent content are 2.80 Å^3^ Da^-1^ and 56.1%, respectively.

Crystals of the heterodimer between Csd1_125–312_ and Csd2_121–308_ were grown at 23°C by the sitting-drop vapor diffusion method. Each sitting drop (0.4 μl) was prepared by mixing equal volumes of the protein solution at 8 mg/ml and the reservoir solution [100 mM Tris-HCl at pH 8.5, and 25% (w/v) PEG3350]. The sitting drop was equilibrated against 100 μl of the reservoir solution. A cluster of needle-like crystals grew up to approximate dimensions of 0.2 mm × 0.01 mm × 0.01 mm in two weeks. We initially tried to optimize this crystallization condition but failed to improve the crystal quality. Therefore, microseeding was employed. A stock solution of microseed crystals was prepared by crushing the needle-like crystals in 50 μL of the reservoir solution using the Seed Bead kit (Hampton Research) and by diluting hundred-fold with the reservoir solution. A cluster of rod-shaped crystals was obtained when we used a reservoir solution consisting of 100 mM HEPES at pH 7.0, and 30% (w/v) Jeffamine ED-2001 and 4 μl of the sitting drop, which was prepared by mixing 2 μl of the protein solution, 1.6 μL of the reservoir solution, and 0.4 μL of the microseed crystal stock solution. The crystals grew up to approximate dimensions of 0.2 mm × 0.05 mm × 0.05 mm within a few days. They were cryoprotected in the reservoir solution supplemented with 10% (v/v) glycerol and were flash-frozen in a nitrogen gas stream at 100 K. Two sets of native data were collected from different crystals to 2.27 Å and 2.35 Å resolutions using the ADSC Q270 CCD detector at the beamline BL-7A of Pohang Light Source, Pohang, Korea. Raw X-ray diffraction data were processed and scaled using the program suit HKL2000 [32].

### Model building and refinement

The structure of Csd2_121–308_ homodimer was determined by molecular replacement utilizing the program MOLREP [33], with domain 3 of an outer-membrane protein NMB0315 from *Neisseria meningitidis* (PDB code 3SLU) as a search model. Domain 3 of NMB0315 shows 24% sequence identity with residues Lys149–Asp259 of Csd2_121–308_. Structures of the heterodimer between Csd1_125–312_ and Csd2_121–308_ were solved by molecular replacement using the refined monomer model of Csd2_121–308_ as a search model. The sequence identity between Csd1_125–312_ and Csd2_121–308_ is 39%. Manual model building was done using the program COOT [34] and the models were refined with the programs REFMAC5 [35], including the bulk solvent correction. A total of 5% of the data was randomly set aside as test data for the calculation of R_free_ [36]. The stereochemistry of the refined models was assessed by MolProbity [37]. Atomic coordinates and structure factors are available from the Protein Data Bank (http://wwpdb.org/) under accession codes 5J1K for the Csd2-Csd2 dimer (Csd2_121–308_ homodimer), and 5J1L and 5J1M for Csd1-Csd2 heterodimer model I and heterodimer model II (Csd1_125–312_-Csd2_121–308_ heterodimer), respectively.

### Identification of Zn^2+^ binding by anomalous diffraction data

To confirm the identity of a metal ion bound to the catalytic site of Csd1, a set of single-wavelength anomalous diffraction (SAD) data was collected at 100 K from a crystal of the complex between Csd1_125–312_ and Csd2_121–308_ using the X-ray wavelength of 1.2826 Å (Zn^2+^ absorption edge) at the beamline 7A of Pohang Light Source. Raw data were processed and scaled using HKL2000 [32]. Anomalous difference maps were calculated using the program FFT of the CCP4i software package [38].

### SEC-MALS

SEC-MALS experiments were performed at 23°C using an HPLC system that was connected with a MALS detector (DAWN HELEOS-II, Wyatt Technology) and a differential refractive index detector (Optilab T-Rex, Wyatt Technology). The samples were two Csd2 constructs [Csd2_121–308_ fused with both N- and C-terminal tags and Csd2_140–251_ fused with a C-terminal tag], and a complex between Csd1_54–312_ (fused with a C-terminal tag) and Csd2_121–308_ (without a fusion tag). A size-exclusion chromatography column (WTC-015S5, Wyatt Technology) was pre-equilibrated with the buffer (20 mM HEPES at pH 7.5 and 300 mM sodium chloride) at a flow rate of 0.5 ml/min and was calibrated using a bovine serum albumin protein standard. Protein samples were injected at a flow rate of 0.5 ml/min. Weight-averaged molar masses were calculated from the elution data using the ASTRA software (Wyatt Technology).

### Equilibrium sedimentation

Equilibrium sedimentation experiments were performed in six-sector cells using a Beckman ProteomeLab XL-A analytical ultracentrifuge for Csd2_121–308_ (fused with both N- and C-terminal tags) and Csd2_140–251_ (fused with a C-terminal tag) in 20 mM HEPES at pH 7.5 and 200 mM sodium chloride at 4°C. The protein samples were measured at two different speeds (30,000 and 35,000 rpms) and two different protein concentrations (3.5 and 5.1 μM for Csd2_121–308_, and 10.1 and 14.5 μM for Csd2_140–251_, respectively). The protein concentrations were calculated using ε_280nm_ = 25,440 and 8,940 M^-1^ cm^-1^ for Csd2_121–308_ and Csd2_140–251_, respectively. All measured data fit well to a reversible monomer-dimer (1x-2x) equilibrium model for both Csd2_121–308_ and Csd2_140–251_. Representative results for Csd2_121–308_ (measured at 35,000 rpm and 5.1 μM protein concentration) and Csd2_140–251_ (measured at 35,000 rpm and 14.5 μM protein concentration) are presented. The monomer-dimer (1x-2x) equilibrium model for Csd2_121–308_ gave the weighted root-mean-square (r.m.s.) error value of 8.52 × 10^−3^ with the *K*_a_ value of 2.03 × 10^5^ M^-1^. The monomer-dimer (1x-2x) equilibrium model for Csd2_140–251_ gave the r.m.s. error value of 9.60 × 10^−3^ with the *K*_a_ value of 2.20 × 10^4^ M^-1^.

## Results

### Csd2_121–308_ forms a dimer in the crystal

**Fig 1:**
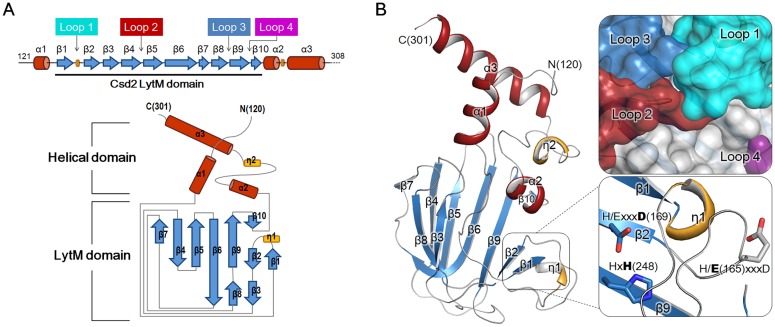
Overall monomer structure of *H*. *pylori* Csd2_121–308_. (A) Schematic representation of secondary structures of Csd2_121–308_ and topology diagram of Csd2_121–308_. Secondary structures have been defined by the STRIDE program [42]. α-Helices, β-strands, 3_10_-helices, and loops are shown as cylinders (colored in red), arrows (skyblue), flat cylinders (yellow), and solid lines (grey), respectively. Loop 1 (β1-β2 loop; cyan), Loop 2 (β4-β5 loop; red), Loop 3 (β8-β9 loop; skyblue), and Loop 4 (β9-β10 loop; purple) form the putative substrate-binding groove of the Csd2 LytM domain. A dotted line indicates the disordered C-terminal region. (B) Ribbon diagram of Csd2_121–308_ monomer structure (chain A of Csd2-Csd2 dimer), colored as in the topology diagram in Fig 1A. Close-up views on the right represent the surface representation of the putative substrate-binding groove formed by four loops of the LytM domain (top) and the degenerated active site without a metal ion (bottom). Close-up views on the right have different orientations from the monomer ribbon diagram on the left to show the details more clearly. Side chains of Glu165, Asp169, and His248, corresponding to the conserved Zn^2+^-coordinating residues, are shown in stick models. Asp169 and His248 belong to the Hxxx**D** and Hx**H** motifs of LytM domains, respectively. In Csd2, Glu165 replaces the histidine residue in the HxxxD motif and it is indicated by the modified H/ExxxD motif. No Zn2+ ion is bound to the Csd2 LytM domain.

The crystal structure of Csd2_121–308_ was determined using a hexagonal crystal (‘Csd2-Csd2 dimer’ in Table 1) with one monomer in the asymmetric unit. The model of Csd2_121–308_ monomer accounting for residues His120–Asp301 was refined at 1.80 Å to R_work_ and R_free_ values of 18.1% and 21.8%, respectively (Table 1). It consists of three α-helices, two 3_10_-helices, and ten β-strands, which are arranged in the order of α1-β1-η1-β2-β3-β4-β5-β6-β7-β8-β9-β10-α2-η2-α3 (Fig 1A). The Csd2_121–308_ monomer can be divided into two structural domains: the helical domain (residues His120–Gly139 and Asp259–Asp301) and the LytM domain (residues Met140–Leu258). The helical domain consists of four helices (α1, α2, η2, and α3) and the LytM domain is inserted between helices α1 and α2 of the helical domain. The LytM domain of Csd2_121–308_ adopts the canonical fold, in which a central seven-stranded anti-parallel β-sheet (β1↑-β2↓-β9↑-β6↓-β5↑-β4↓-β7↑) forms a two-layered sandwich with a smaller anti-parallel β-sheet (β3↓-β8↑-β6↓) (Fig 1B). The long, highly curved strand β6 is shared between these two sheets. A short strand β10 forms a mini, anti-parallel β-sheet (β9↑-β10↓) with the C-terminal side of β9 of the central β-sheet. A DALI search with the Csd2 LytM domain reveals that it exhibits high structural similarity with other LytM domains of the M23 peptidase family, with Z-scores up to 16.7 (S1 Table). Essentially identical results were obtained when the whole chain of Csd2_121–308_ was used for the DALI search. The helical domain of Csd2_121–308_ is structurally unique.

**Table 1:** Data collection and refinement statistics.

**Data collection**				
Data set	Csd2-Csd2 dimer	Csd1-Csd2 dimer I	Csd1-Csd2 dimer II	Zn SAD
Space group	P6_1_22	P2_1_	P2_1_	P2_1_
*a*, *b*, *c* (Å)	140.8, 140.8, 40.2	53.4, 82.4, 76.3	53.1, 80.0, 74.6	53.5, 80.4, 74.6
α, β, γ (°)	90, 90, 120	90, 106.0, 90	90, 104.4, 90	90, 104.7, 90
X-ray wavelength (Å)	0.97960	0.97933	0.97933	1.2826
Resolution range (Å)	50.0–1.80	50.0–2.27	50.0–2.35	50.0–2.90
(highest resolution shell)	(1.83–1.80)^a^	(2.31–2.27)^a^	(2.39–2.35)^a^	(2.95–2.90)^a^^,^^b^
Total / unique reflections	365,382 / 22,028	118,925 / 29,291	155,055 / 25,521	122,620 / 13,536^b^
Completeness (%)	99.8 (98.6)^a^	99.8 (100.0)^a^	99.9 (100.0)^a^	98.6 (87.6)^a^^,^^b^
*<I>* / <*σ*_*I*_*>*	47.1 (6.9)^a^	20.8 (4.0)^a^	26.6 (3.9)^a^	49.7 (21.5)^a^^,^^b^
R_merge_ ^c^ (%)	12.8 (75.1)^a^	13.5 (73.0)^a^	13.7 (88.6)^a^	16.7 (72.1)^a^^,^^b^
R_rim_^d^ (%)	13.2 (77.4)^a^	15.5 (83.6)^a^	15.0 (96.5)^a^	17.8 (76.1)^a^^,^^b^
R_pim_^e^ (%)	3.2 (18.6)^a^	7.6 (40.3)^a^	6.0 (37.9)^a^	6.0 (24.2)^a^^,^^b^
CC_1/2_ ^f^ (%)	99.8 (94.1)^a^	99.3 (70.1)^a^	99.5 (75.9)^a^	99.3 (95.4)^a^^,^^b^
**Model refinement**				
PDB code	5J1K	5J1L	5J1M	-
Resolution range (Å)	50.0–1.80	50.0–2.27	50.0–2.35	-
R_work_ / R_free_^g^ (%)	18.1 / 21.8	17.4 / 23.3	16.8 / 23.3	-
No. of non-hydrogen atoms / average *B*-factor (Å^2^)				
Protein	1,457 / 22.5	5,432 / 32.8	5,418 / 45.0	-
Water oxygen	97 / 26.8	160 / 30.0	140 / 40.0	-
Glycerol	12 / 51.5	-	-	-
Zn^2+^ ion	-	2 / 63.0	2 / 59.3	-
R.m.s. deviations from ideal geometry				
Bond lengths (Å)	0.011	0.010	0.010	-
Bond angles (°)	1.43	1.47	1.40	-
R.m.s. Z-scores				
Bond lengths (%)	54.9	51.6	49.6	-
Bond angles (%)	67.6	68.4	65.1	-
Ramachandran plot^h^				
Favored / outliers (%)	97.2 / 0.0	95.2 / 0.0	95.1 / 0.0	-
Poor rotamers (%)	0.63	0.68	0.68	-

^*a*^ Values in parentheses refer to the highest resolution shell.

^*b*^ Friedel pairs were treated as separate observations.

^*c*^ R_merge_ = Σ_h_Σ_i_ | *I*(*h*)_i_−<*I*(*h*)> | / Σ_h_Σ_i_ *I*(*h*)_i_, where *I*(*h*) is the intensity of reflection *h*, Σ_h_ is the sum over all reflections, and Σ_i_ is the sum over i measurements of reflection *h*.

^*d*^ R_rim_ = Σ_hkl_{*N*(*hkl*) / [*N*(*hkl*)– 1]}^1/2^ Σ_i_ | *I*_i_(*hkl*)–<*I*(*hkl*)> | / Σ_hkl_Σ_i_ *I*_i_(*hkl*). The redundancy-independent merging R factor gives the precision of individual intensity [39].

^*e*^ R_pim_ = Σ_hkl_{1 / [*N*(*hkl*)– 1]}^1/2^ Σ_i_ | *I*_i_(*hkl*)–<*I*(*hkl*)> | / Σ_hkl_Σ_i_ *I*_i_(*hkl*). The precision indicating merging R factor describes the precision of the averaged intensity [40].

^*f*^ CC_1/2_ is the correlation coefficient of the mean intensities between two random half-sets of data [41].

^*g*^ R_work_ = Σ | |*F*_obs_|–|*F*_calc_| | / Σ |*F*_obs_|, where R_free_ is calculated for a randomly chosen 5% of reflections, which were not used for structure refinement and R_work_ is calculated for the remaining reflections.

^*h*^ Values obtained using MolProbity.

**Fig 2:**
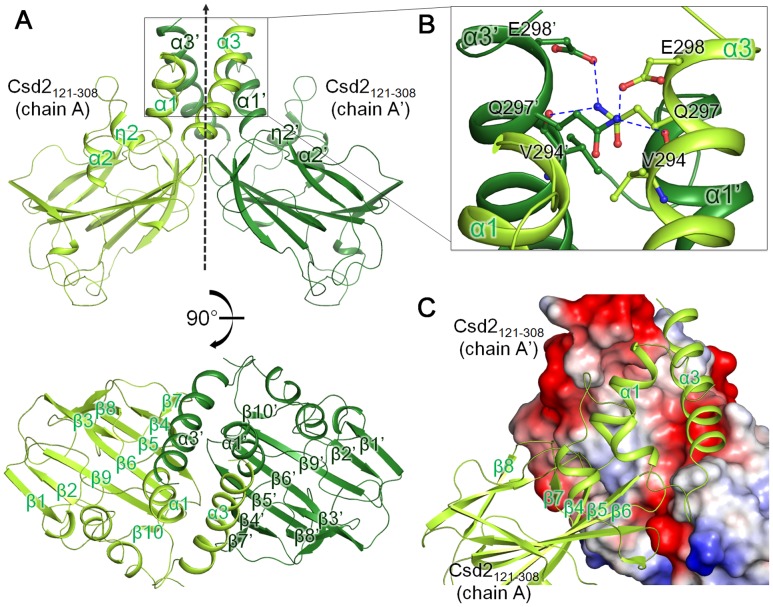
*H*. *pylori* Csd2_121–308_ exists as homodimer in the crystal. (A) Two different views of the *H*. *pylori* Csd2_121–308_ homodimer structure are shown in ribbon diagram. One Csd2_121–308_ monomer (chain A colored in yellow-green) and the other Csd2_121–308_ monomer (chain A’ colored in darker green) from the adjacent asymmetric unit form a homodimer around a crystallographic two-fold symmetry axis (indicated by a dotted arrow) in the crystal. The secondary structure elements of the helical domain are labeled in the side view (top), while most of the secondary structures are labeled in the top view (bottom). (B) Close-up view of the dimer interface. Residues at the dimer interface are shown in stick models. Blue dotted lines represent hydrogen bonds. (C) The interface between Csd2_121–308_ monomers. One Csd2_121–308_ monomer (chain A) is shown as a ribbon diagram (in yellow-green) and the other Csd2_121–308_ monomer (chain A’ from the adjacent asymmetric unit) is shown in the electrostatic surface diagram. This view of the dimer is slightly different from the top view in Fig 2A to show the details more clearly. The dimer interface is hydrophobic in the center and is surrounded by negatively charged surfaces.

Unexpectedly, the Csd2_121–308_ monomer (referred to as chain A) in the asymmetric unit of the crystal forms a tight symmetric side-by-side homodimer with a neighboring Csd2_121–308_ monomer (chain A’) from an adjacent asymmetric unit (Fig 2A). In this homodimer of Csd2_121–308_, a surface area of 1,550 Å^2^ per monomer is buried at the interface, as analyzed by the PISA server [43]. The pair of helices α1 and α3 from one helical domain of Csd2_121–308_ pack against another pair of helices (α1’ and α3’) from the helical domain of another Csd2_121–308_ in the adjacent asymmetric unit to form a four-helix bundle through hydrophobic and hydrogen-bond interactions around a crystallographic two-fold symmetry axis (Fig 2A). At the interface between helix pairs, the side chain of Gln297 forms hydrogen bonds with the main-chain oxygen of Val294 and the side chain of Glu298 (Fig 2B). Numerous hydrophobic side chains are present at the interface, which are distributed in both the helix pair and the LytM domain (β4, β5-β6 loop, β6-β7 loop, β7-β8 loop, and β9-β10 loop) (Fig 2C).

### Csd2 exists as monomer-dimer equilibrium in solution

**Fig 3:**
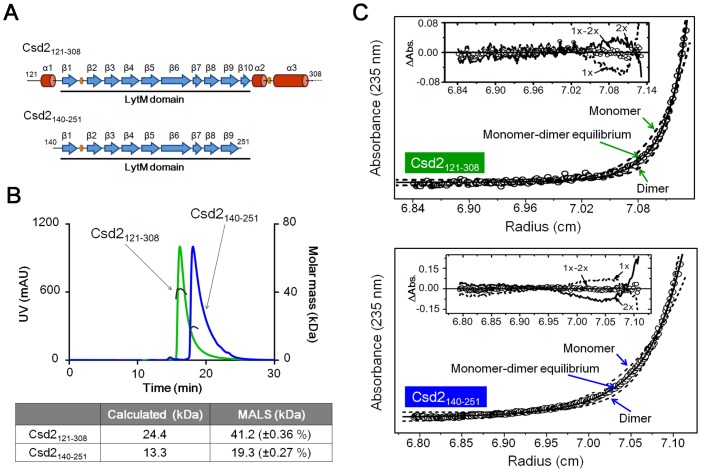
SEC-MALS and equilibrium sedimentation experiments to determine the oligomeric state of *H*. *pylori* Csd2 in solution. (A) Two Csd2 constructs (Csd2_121–308_ and Csd2_140–251_) used in these experiments are schematically represented with the secondary structure elements colored as in Fig 1A. Csd2_140–251_ lacks the helical domain. Csd2_121–308_ was used for structure determination. (B) SEC-MALS data for two Csd2 protein samples. The black solid lines represent the measured molecular masses. The average molecular masses from MALS analyses are compared with the calculated masses in the table below the chromatography profiles. (C) Equilibrium sedimentation data for two Csd2 protein samples. For Csd2_121–308_ (top), the circles are experimental data measured at a speed of 35,000 rpm and 5.1 μM protein monomer concentration and the solid line is a fitting line for a reversible monomer-dimer equilibrium model. The two dotted lines are fitting lines for ideal homogeneous monomer and dimer models. Distributions of the residuals for monomer (dotted line), dimer (solid line), and reversible monomer-dimer equilibrium (circles) models are shown in the inset panel. For Csd2_140–251_ (bottom), the circles are experimental data measured at a speed of 35,000 rpm and 14.5 μM protein monomer concentration and the solid line is a fitting line for a reversible monomer-dimer equilibrium model. The two dotted lines are fitting lines for ideal homogeneous monomer and dimer models. Distributions of the residuals for monomer (dotted line), dimer (solid line), and reversible monomer-dimer equilibrium (circles) models are shown in the inset panel. Equilibrium sedimentation data indicate that both Csd2_121–308_ and Csd2_140–251_ are in reversible monomer-dimer equilibrium in solution.

Since Csd2_121–308_ was found to exist as a homodimer in the crystal, we investigated the oligomeric state of Csd2 (using Csd2_121–308_ and Csd2_140–251_) in solution by both SEC-MALS and equilibrium sedimentation experiments. The shorter Csd2_140–251_ construct covers most of the LytM domain except the short β10 strand and lacks the entire helical domain (Fig 3A). Molecular masses estimated by SEC-MALS are 41.2 and 19.3 kDa, respectively, for Csd2_121–308_ (with the calculated molecular mass of 24.4 kDa, including the N-terminal and C-terminal fusion tags) and Csd2_140–251_ (with the calculated molecular mass of 13.3 kDa, including the C-terminal fusion tag) (Fig 3B). The measured masses are larger than the calculated mass of monomeric species, but are smaller than the calculated mass of dimeric species for both constructs of Csd2, making it difficult to assign unambiguously the oligomeric state of these Csd2 proteins in solution.

Therefore, we additionally carried out equilibrium sedimentation experiments. The data measured at two different rotor speeds and at two different protein concentrations indicate that both Csd2_121–308_ and Csd2_140–251_ exist in reversible monomer-dimer (1x-2x) equilibrium in solution. The representative results, as shown in Fig 3C, gave dissociation constants (*K*_d_) of 4.9 μM for Csd2_121–308_ and 45 μM for Csd2_140–251_. Deletion of the helical domain was not sufficient for complete disruption of Csd2_140–251_ dimerization, but resulted in facilitated dissociation of Csd2_140–251_ dimers, with about a nine-fold increase in the *K*_d_ value. This finding is in agreement with the crystal structure of Csd2_121–308_ homodimer, in which the helical domain makes up a large portion of the dimer interface but not all. On the basis of these results, one may expect that Csd2 can possibly interact with its close homolog such as Csd1 in a similar manner. We confirmed by SEC-MALS that Csd2_121–308_ and Csd1_54–312_ form a stable heterodimer, as described in detail below.

### Csd1 and Csd2 can form a stable heterodimer in solution

**Fig 4:**
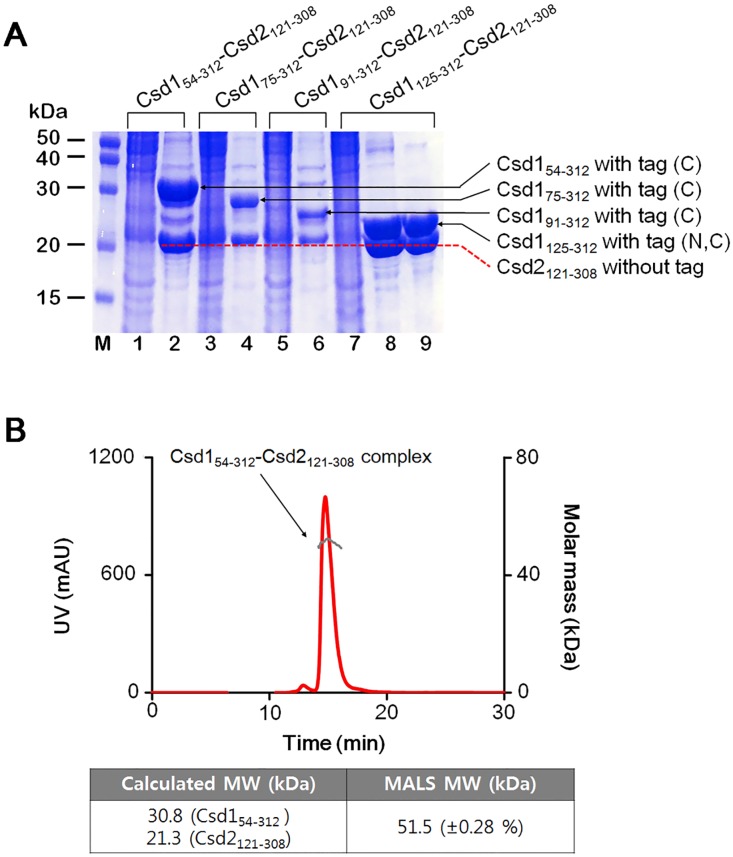
Analysis of the complex formation between *H*. *pylori* Csd1 and Csd2. (A) SDS-PAGE analysis of the Csd1 and Csd2 complex formation. Lane M: pre-stained protein ladder. Lanes 1 and 2 for Csd1_54-312_-Csd2_121-308_ complex: pooled fractions either unbound (lane 1) or bound (lane 2) to the affinity chromatography. Lane 3 (unbound) and lane 4 (bound) for Csd1_75-312_-Csd2_121-308_ complex. Lane 5 (unbound) and lane 6 (bound) for Csd1_91-312_-Csd2_121-308_ complex. Lane 7 (unbound) and lane 8 (bound) for Csd1_125-312_-Csd2_121-308_ complex. Lane 9: the peak fraction of the Csd1_125-312_-Csd2_121-308_ complex after size exclusion chromatography. This complex was crystallized for structure determination. (B) SEC-MALS data for the Csd1_54–312_-Csd2_121–308_ complex. The red line represents the size exclusion chromatography profile. The grey line represents the measured molecular mass, whose average value agrees well with the calculated molecular mass of a 1:1 complex, as shown in the table below the chromatography profile.

To examine the possible complex formation between Csd1 and Csd2 by affinity chromatography, we overproduced four Csd1 constructs fused with one or two hexahistidine-containing tags and Csd2_121–308_ without a fusion tag. We then purified four possible complexes between Csd1 and Csd2 by mixing the cell pellets that individually express either a Csd1 construct or the Csd2_121–308_ construct, as described in Materials and Methods. The tested Csd1 constructs are (i) Csd1_54–312_ fused with a C-terminal tag, (ii) Csd1_75–312_ with a C-terminal tag, (iii) Csd1_91–312_ with a C-terminal tag, and (iv) Csd1_125–312_ with tags at both N- and C-termini. The complex formation between each of the above four Csd1 constructs and Csd2_121–308_ could be readily identified by SDS-PAGE analysis (Fig 4A). This result indicates that stable complexes of different Csd1 constructs and Csd2_121–308_ can be formed and purified, when individually expressed Csd1 and Csd2 proteins are present in roughly equal amounts. This implies that these Csd1-Csd2 complexes are more stable than the Csd2_121–308_ homodimer.

To establish the stoichiometry of the Csd1-Csd2 complexes, we estimated the molecular mass of the purified complex between Csd1_54–312_ and Csd2_121–308_ by SEC-MALS (Fig 4B). The measured molecular mass of 51.5 kDa for the complex agrees well with the calculated total mass of Csd1_54–312_ (30.8 kDa) and Csd2_121–308_ (21.3 kDa). This result establishes that Csd1_54–312_ and Csd2_121–308_ bind in a 1:1 molar ratio. Additionally, we have determined the crystal structure of the 1:1 complex between Csd1_125–312_ and Csd2_121–308_, as described in more detail below.

### Crystal structure of the heterodimer between Csd1_125–312_ and Csd2_121–308_

**Fig 5:**
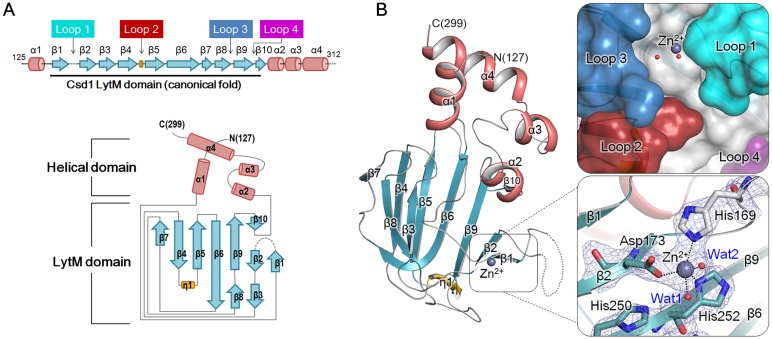
Overall monomer structure of *H*. *pylori* Csd1_125–312_. (A) Schematic representation of secondary structures of Csd1_125–312_ and topology diagram of Csd1_125–312_. Secondary structures have been defined by the STRIDE program [42]. α-Helices, β-strands, 3_10_-helices, and loops are shown as cylinders (colored in light pink), arrows (blue-green), flat cylinders (yellow), and solid lines (grey), respectively. Loop 1 (β1-β2 loop; cyan), Loop 2 (β4-β5 loop; red), Loop 3 (β8-β9 loop; skyblue), and Loop 4 (β9-β10 loop; purple) form the substrate-binding groove of the Csd1 LytM domain. Dotted lines indicate disordered regions. (B) Ribbon diagram of Csd1_125–312_ monomer structure (chain C of Csd1-Csd2 dimer I), colored as in the topology diagram in Fig 5A. Close-up views on the right represent the surface representation of the substrate-binding groove formed by four loops of the LytM domain (top) and canonical Zn^2+^-coordination with three protein ligands and two water molecules (bottom). Dark grey and red spheres represent a Zn^2+^ ion and water molecules, respectively. Side chains of the Zn^2+^-coordinating residues (His169, Asp173, and His252) are shown in stick models. Black dotted lines denote penta-coordination of the Zn^2+^ ion. The electron density for the Zn^2+^-bound active site in 2mF_o_ − DF_c_ map (grey colored mesh) are shown at the 1.0 *σ* level.

To reveal detailed interactions between Csd1 and Csd2, we purified four complexes (Csd1_54–312_-Csd2_121–308_, Csd1_75–312_-Csd2_121–308_, Csd1_91–312_-Csd2_121–308_, and Csd1_125–312_-Csd2_121–308_) and tried to crystallize them. Only the Csd1_125–312_-Csd2_121–308_ complex was crystallized in the monoclinic P2_1_ space group. We have solved the structure of this complex using two different sets of data collected from two different crystals at 2.27 and 2.35 Å resolutions, respectively (Csd1-Csd2 dimer I and Csd1-Csd2 dimer II in Table 1). In both heterodimer models, two copies of the heterodimer (AB dimer and CD dimer) are present in the asymmetric unit, with each heterodimer consisting of Csd1_125–312_ (chain A or C) and Csd2_121–308_ (chain B or D) in a 1:1 molar ratio (S1 Fig). The two heterodimers within the asymmetric unit are related to each other by non-crystallographic two-fold symmetry, with Cα r.m.s. deviations of 1.86 Å and 2.15 Å for 332 and 334 residues for heterodimer models I and II, respectively. Csd1_125–312_ and Csd2_121–308_ chains within each of the heterodimers are related by pseudo two-fold symmetry due to their overall structural similarity (Cα r.m.s. deviations of 1.95–4.47 Å for 159–165 residues). The Csd1_125–312_ and Csd2_121–308_ structures in dimer model I account for the following residues: (i) 129–153 and 165–299 of chain A, (ii) 127–156 and 168–299 of chain C, (iii) 123–304 of chain B, and (iv) 123–297 of chain D. The Csd1_125–312_ and Csd2_121–308_ structures in dimer model II account for the following residues: (i) 128–154 and 161–299 of chain A, (ii) 128–154 and 169–300 of chain C, (iii) 122–297 of chain B, and (iv) 122–298 of chain D. The C-terminal residues 299–304 of Csd2_121–308_ (chain B) in dimer model I are not involved in hetero-dimerization, but are instead inserted into the substrate binding groove of Csd1_125–312_ (chain C’), as described in more detail below. The Csd1_125–312_ monomer (chain C in heterodimer model I) is comprised of four α-helices, one 3_10_-helix, and ten β-strands in the order α1-β1-β2-β3-β4-η1-β5-β6-β7-β8-β9-β10-α2-α3-α4 (Fig 5A). Similarly to Csd2_121–308_, the structure of Csd1_125–312_ can be divided into two domains: a helical domain (residues Ile127–Asp142 and Asn263–Gln299) consisting of four helices (α1–α4) and the C-terminal LytM domain (residues Tyr143–Ile262) consisting of ten strands (β1–β10) (Fig 5). As in Csd2_121–308_, the C-terminal LytM domain is inserted between helices α1 and α2 of the helical domain.

A strong electron density was observed at the metal-binding site in each of Csd1_125–312_ chains A and C in both heterodimer models I and II (S1 Fig). As the key residues for the catalytic activity are well conserved in the Csd1 LytM domain, we have assigned the metal ion as Zn^2+^. This assignment was confirmed by the anomalous difference electron density maps calculated using the anomalous data collected to 2.90 Å at the Zn^2+^ X-ray absorption edge of 1.2826 Å from a third P2_1_ crystal (Table 1). In contrast, no electron density is observed at the corresponding site of Csd2_121–308_ chains B and D in both heterodimer models I and II (S1 Fig), indicating that Zn^2+^ is not bound.

All four Csd2_121–308_ structures (chains B and D) in the two heterodimer models are essentially identical, with Cα r.m.s. deviations of 0.29–0.42 Å for 175–176 residues. They also do not differ much from the Csd2_121–308_ structure in the homodimer model, with Cα r.m.s. deviations of 0.44–1.05 Å for 175–179 residues. Interestingly, however, the segments of Csd1_125–312_ around the metal-binding site display diverse structures among different chains of Csd1_125–312_ in the two heterodimer models. More specifically, four Csd1_125–312_ structures (chains A and C) in the two heterodimer models adopt three different modes of metal coordination in the LytM domain (S2A Fig). Two Csd1_125–312_ structures in dimer model I (chain C) and model II (chain C) are virtually identical with a Cα r.m.s. deviation of 0.44 Å for 158 residues and share the canonical metal coordination. Two Csd1_125–312_ structures in dimer model I (chain A) and model II (chain A) deviate from chains C (Cα r.m.s. deviations of 2.60–3.04 Å for 156–159 residues) but they also differ from each other (Cα r.m.s. deviation of 2.04 Å for 160 residues). As a consequence, we observe two different types of non-canonical metal-coordination by the Csd1 LytM domain (S2B Fig), as further discussed below. The observed structural differences around the metal-binding sites of Csd1_125–312_ have little effect on the heterodimerization pattern, because the metal-binding site is well separated from the interface between Csd1_125–312_ and Csd2_121–308_ in the heterodimer.

**Fig 6:**
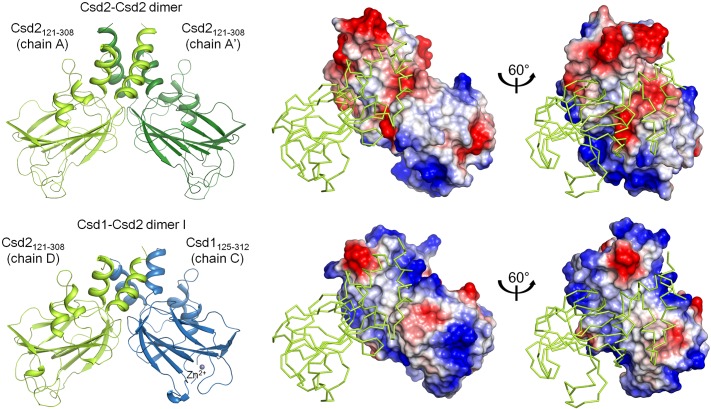
Comparison of Csd2_121–308_ homodimer and Csd1_125–312_-Csd2_121–308_ complex (heterodimer I). Csd2_121–308_ homodimer (top left) and the Csd1_125–312_-Csd2_121–308_ complex (heterodimer I) (bottom left) are shown in ribbon diagrams; they are colored as in Fig 2A and S1 Fig, respectively. Electrostatic surface diagrams of Csd2_121–308_ chain A’ of Csd2 homodimer and Csd1_125–312_ chain C of the Csd1_125–312_-Csd2_121–308_ heterodimer are shown on the right. Highly negatively-charged surfaces surround the hydrophobic interface of the Csd2_121–308_ homodimer, whereas largely positively-charged surfaces surround the hydrophobic interface of Csd1_125–312_ of the Csd1_125–312_-Csd2_121–308_ heterodimer.

In the crystal structure of the heterodimer between Csd1_125–312_ and Csd2_121–308_, the pair of helices α1 and α4 from the helical domain of Csd1_125–312_ pack against the corresponding pair of helices α1 and α3 from the helical domain of Csd2_121–308_ to form a four helix bundle, in which one Csd1_125–312_ monomer essentially replaces a Csd2_121–308_ monomer within the Csd2_121–308_ homodimer (Fig 6). Buried surface areas per monomer of Csd1_125–312_ and Csd2_121–308_, as calculated using the PISA sever [43], differ by only 1.1–4.4%. Four heterodimers have buried surface areas per monomer (averaged over Csd1_125–312_ and Csd2_121–308_) of 1,310, 1,290, 1,400, and 1,440 Å^2^ for AB and CD heterodimer models I and II, respectively. These values are slightly smaller than that for the Csd2_121–308_ homodimer, because more N- and C-terminal residues of Csd2_121–308_ as well as Csd1_125–312_ are disordered and invisible in the heterodimers between Csd1_125–312_ and Csd2_121–308_ than in the Csd2_121–308_ homodimer (Fig 6). An extended hydrophobic region is found around the center of the dimer interface in both the Csd2_121–308_ homodimer and the heterodimer between Csd1_125–312_ and Csd2_121–308_ (Fig 6). However, surface-charge distributions of the surrounding regions in Csd1_125–312_ and Csd2_121–308_ are strikingly different. Highly negatively charged surfaces surround the hydrophobic interface of the Csd2_121–308_ homodimer, whereas largely positively charged surfaces surround the hydrophobic interface of Csd1_125–312_ of Csd1_125–312_ and Csd2_121–308_ heterodimer (Fig 6). The interface between Csd1_125–312_ and Csd2_121–308_ harbors a network of hydrogen bonds and a salt bridge (S3A Fig). Asp292 on helix α3 of Csd2_121–308_ forms a salt bridge with Arg137 on helix α1 of Csd1_125–312_ and His130 on helix α1 of Csd2_121–308_ hydrogen bonds with Gln299 on helix α4 of Csd1_125–312_ (S3B Fig). A hydrogen bond is formed between the side chains of Gln288 of Csd2_121–308_ and Arg296 of Csd1_125–312_. The main chain of Lys224 of Csd2_121–308_ is hydrogen bonded to Arg286 of Csd1_125–312_. These hydrogen bonds are formed between strand β7 of Csd2 LytM domain and helix α4 of Csd1 helical domain (S3C Fig). Two arginine residues (Arg286 and Arg296) on helix α4 of Csd1_125–312_ correspond to negatively charged residues Glu282 and Asp292 of Csd2_121–308_, respectively (S3D Fig).

### LytM domains of Csd1 and Csd2

Amino-acid sequences of Csd1, Csd2, and Csd3 from *H*. *pylori* 26695 strain are well aligned over their LytM domains, whose overall structures are also similar to each other. Two characteristic motifs (HxxxD and HxH) of the LytM domain are conserved in Csd1 and Csd3, whereas the first histidines of these motifs in Csd2 are substituted with Glu165 and Lys246, respectively (S4A Fig). Csd2 may possess a degenerate active site in its LytM domain, like other non-peptidase members of the M23B metallopeptidase family. In addition, no metal ion is bound to the active site in the Csd2 LytM domain in structures of both Csd2 homodimer and Csd1-Csd2 heterodimer (Fig 1B and S1 and S4B Figs). Despite the absence of a metal ion in the active site, the Csd2 LytM domain possesses a substrate-binding groove formed by four loops (Loop 1; β1-β2, Loop 2; β4-β5, Loop 3; β8-β9, and Loop 4; β9-β10) on the central β-sheet (Fig 1B and S4B Fig). Loop 1 is folded over the degenerate active site and covers it. Similar substrate-binding sites are present in other M23 family member proteins [44].

The four Csd1_125–312_ monomers in two heterodimer models I and II show three different modes of metal-coordination in the LytM domain: the canonical metal-coordination (for both chains C in heterodimer models I and II) and two other non-canonical modes of metal-coordination (i.e., non-canonical coordination A for chain A of heterodimer model I and non-canonical coordination B for chain A of heterodimer model II) (S2 Fig). The observed diversity in metal-coordination by the LytM domains of four Csd1_125–312_ monomers in two heterodimer models I and II, likely resulting from differences in their environment within the crystals, suggests that the segment of Csd1_125–312_ covering residues Leu145–Leu172 possesses high flexibility and structural plasticity in forming the metal-binding site. This segment includes strand β1 and Loop 1 (S2A Fig). In the structure of Csd3 LytM domain [30], the floor of the substrate-binding groove is built upon the central β-sheet and the walls of the active site are made up of four loops: Loop I (β12-β13 loop), Loop II (β15-β16 loop), Loop III (β19-β20 loop), and Loop IV (β20-β21 loop). In Form 1 crystals of Csd3, Loop I is involved in crystal packing interactions by forming sulfate-mediated salt bridges. In Form 2 crystals, it is not involved in the crystal packing and is disordered. Therefore, it was concluded that the observed structural variation of Loop I is largely due to the difference in crystal packing and also due to its inherent flexibility [30]. Despite the flexibility of Loop I in Csd3, only the canonical Zn-coordination was observed.

In the LytM domain of Csd1_125–312_ (chain C of heterodimer model I), with the canonical Zn^2+^-coordination, a central anti-parallel β-sheet (β1↑-β2↓-β9↑-β6↓-β5↑-β4↓-β7↑) forms a two-layered sandwich with a smaller anti-parallel β-sheet (β3↓-β8↑-β6↓) (Fig 5B and S4B Fig). The long, highly curved strand β6 is shared between these two sheets. A short strand β10 forms a mini, anti-parallel β-sheet (β9↑-β10↓) with the C-terminal side of β9 of the central β-sheet. As expected, the Csd1 LytM domain exhibits a high level of structural similarity with other LytM domains of the M23 peptidase family, with Z-scores up to 18.5 (S2 Table). The central β-sheet anchors the catalytic residues, which are grouped around the Zn^2+^ ion. The zinc-bound active site is located in a substrate-binding groove that is made up of four loops (Loop 1; β1-β2, Loop 2; β4-β5, Loop 3; β8-β9, and Loop 4; β9-β10) (Fig 5B and S4B Fig). In the LytM domain of Csd1_125–312_, Loop 1 is partially disordered and Loop 2 contains a 3_10_-helix (η1). The Zn^2+^ ion is penta-coordinated in slightly distorted trigonal bipyramidal geometry with the expected three ligands (His169 and Asp173 of the **H**xxx**D** motif and His252 of the Hx**H** motif) and two water molecules (Fig 5B and S2B Fig). The metal-ligand atom distances are in the range of 2.0–2.5 Å, consistent with typical Zn^2+^ ion-ligand atom distances. The two water molecules are assigned as Wat1 (Wat1–Zn^2+^ 2.5 Å), which is coordinated by conserved histidine residues (His 250 of the **H**xH motif and His219), and Wat2 (Wat2–Zn^2+^ 2.3 Å). The observed penta-coordination is coincident with a catalytic mechanism proposed for LasA from *Pseudomonas aeruginosa* [44] and other LytM domains [45]. In the tartrate-uncomplexed structure of LasA from *P*. *aeruginosa*, the Zn^2+^ ion is also penta-coordinated by three conserved metal ligands and two water molecules. The two water molecules are assigned as Wat1 (Wat1–Zn^2+^ 2.1 Å), which is oriented by interactions with two conserved histidine residues (His120 of the **H**xH motif and His80), and Wat2 (Wat2–Zn^2+^ 2.7 Å) [44]. In the proposed mechanism, the substrate carbonyl oxygen displaces Wat2, while Wat1 functions as a nucleophile to attack the polarized carbonyl bond [44,45]. In the inactivated structure of Csd3 [30], the Zn^2+^ ion in the active site of the LytM domain is tetra-coordinated, with His259 and Asp263 of the **H**xxx**D** motif, His341 of the Hx**H** motif, and Glu74 from the α3 helix of Domain 1. Two water molecules necessary for the peptidase activity are replaced by side-chain oxygens from Glu74: Wat1 by Glu74^ε2^ (2.0 Å from the Zn^2+^ ion) and Wat2 by Glu74^ε1^ (2.9 Å from the Zn^2+^ ion).

Two other non-canonical modes of Zn^2+^-coordination in the Csd1_125–312_ structures of heterodimer models I and II have not been reported previously. In both non-canonical coordination A and B, one of the three metal ligands (His169 of the HxxxD motif) does not participate in the Zn^2+^-coordination and an additional α-helix (α1a) is formed in the linker between α-helix α1 of the helical domain and β-strand β1 of the LytM domain (S2A Fig), making Loop 1 shorter than in the canonical coordination. However, residues between helix α1a and Loop 1 are disordered in the crystal. In the non-canonical coordination A, the Zn^2+^ ion has a high B-factor of 82.4 Å^2^, possibly due to partial occupancy. Thus, the Zn^2+^-ligand atom distances are longer than usual (2.6 Å and 3.0 Å to Asp173 of the Hxxx**D** motif, 2.9 Å to His250 of the **H**xH motif, and 3.3 Å to His252 of the Hx**H** motif, and 3.3 Å to His219) (S2B Fig). In the non-canonical coordination B, the Zn^2+^ ion is tetrahedrally coordinated and has a lower B-factor of 43.8 Å^2^, suggesting full occupancy. The Zn^2+^-ligand atom distances are usual (1.9 Å to Asp173 of the Hxxx**D** motif, 2.2 Å to His252 of the Hx**H** motif, 2.4 Å to His164, and 2.1 Å to Wat1) (S2B Fig). In Csd1, the Zn^2+^-coordinating His164 extends the HxxxD motif, resulting in the H_(164)_xxxxH_(169)_xxxD_(173**)**_. His164 is not conserved in *H*. *pylori* Csd2 and Csd3. This may explain why non-canonical coordination is not observed for Csd2 and Csd3.

### The C-terminal tail of Csd2 occupies the substrate-binding groove of Csd1 LytM domain in the crystal

**Fig 7:**
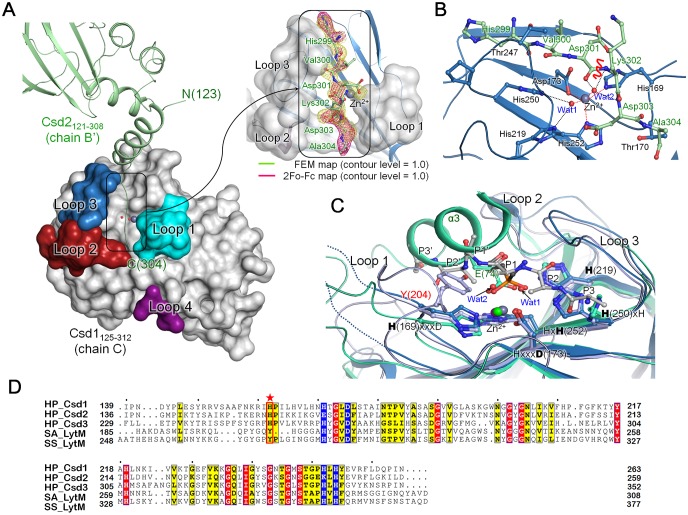
The C-terminal tail of Csd2 (chain B’) is bound to the substrate-binding groove in the LytM domain of Csd1 (chain C) in Csd1-Csd2 dimer I. (A) In the structure of Csd1-Csd2 dimer I, the C-terminal residues (His299*−*Ala304) of Csd2_121–308_ from an adjacent asymmetric unit (chain B’ shown in ribbon diagram) occupy the substrate-binding groove of the LytM domain in Csd1_125–312_ (chain C shown in surface diagram). Four loops of Csd1 LytM domain that form the substrate-binding groove are labeled and colored as in Fig 5. The ribbon diagram is colored as in S1 Fig A close-up view on the right represents the Csd2 C-terminal tail residues located in the substrate-binding groove of Csd1 LytM domain. The Csd2 tail residues (enclosed in the black box) are shown in a stick model, with the electron density shown in mesh. The electron density for the Csd2 tail in the feature-enhance map (FEM) calculated by using PHENIX program [47] (lime colored mesh) and 2mF_o_ − DF_c_ map (magenta colored mesh) are shown at the 1.0 *σ* level. (B) A detailed view of the interactions between the Csd2 tail residues and the substrate-binding groove of the Csd1 LytM domain (shown in ribbon diagram, colored as in S1 Fig). Both main chains and side chains of the Csd2 tail residues are shown in a stick model, with the candidate peptide bond that might be cleaved by the enzymatic activity of Csd1 is indicated by a red wavy line. Side chains of Csd1_125–312_ residues interacting with the Csd2 tail residues are shown in a stick model. Grey and red spheres represent the Zn^2+^ ion and water molecules, respectively. Zn^2+^-coordination (canonical) and hydrogen bonds with waters are indicated by red and black dotted lines, respectively. (C) Superposition of LytM domains in *H*. *pylori* Csd1 (skyblue), *H*. *pylori* Csd3 (light green; PDB code, 4RNY), and *S*. *aureus* LytM bound with tetraglycine phosphinate (purple; PDB code, 4ZYB) shows that the two water molecules (Wat1 and Wat2) of Csd1_125–312_ chain C of heterodimer I overlap nicely with side chain oxygen atoms of Glu74 (labeled in light green) from the helix α3 of the inhibitory Domain 1 in Csd3 and also with those of the phosphinate molecule (black). The Csd2 tail is simplified as a poly-alanine model (grey). The bound Zn^2+^ ions are indicated by grey, purple, and green spheres for *H*. *pylori* Csd1, *H*. *pylori* Csd3, and *S*. *aureus* LytM, respectively. Two dotted lines represent the disordered regions in Loop 1 of Csd1. The metal-coordinating residues in the **H(169)**xxx**D(173)** and Hx**H(252)** motifs and the conserved catalytic residues in the **H(250)**xH motif and an additional catalytic histidine residue **H(219)** of Csd1, as well as corresponding residues of *H*. *pylori* Csd3 and *S*. *aureus* LytM, are shown in a stick model. Tyr204 (labeled in red) of *S*. *aureus* LytM is shown in a stick model. (D) Sequence alignment of LytM domains in Csd1, Csd2, and Csd3 from *H*. *pylori* 26695 strain [Csd1 (HP_Csd1; SWISS-PROT accession code O26068), Csd2 (HP_Csd2; O26069), and Csd3 (HP_Csd3; O25247)], *S*. *aureus* LytM (SA_LytM; O33599), and *S*. *simulans* lysostaphin (SS_LytM; P10547). Tyr204 of *S*. *aureus* LytM is marked by a red star. Conserved residues of the characteristic motifs are colored in blue.

Interestingly, during refinement of the heterodimer model I, we observed an extra electron density that extends from the C-terminal residue Glu298 of Csd2_121–308_ (chain B’) from an adjacent asymmetric unit of the crystal and runs along the groove between Loops 1 and 3 of active site of Csd1_125–312_ (chain C with the canonical Zn^2+^-coordination) (Fig 7A). We modeled this electron density as the C-terminal tail sequence HVDKDA of Csd2_121–308_, encompassing residues 299–304 (Fig 7A). The electron density is weaker in the middle of the sequence HVDKDA. A similar electron density is absent in the active site of other Csd1_125–312_ chains of heterodimer models I and II. This may be due to the fact that the HVDKDA sequence differs from the sequence of the physiological substrate of Csd1. Nevertheless, the observed binding of the Csd2 tail sequence in the active site of Csd1 may define the substrate-binding site (P3-P2-P1-P1’-P2’-P3’) of the Csd1 LytM domain, because the central peptide bond between Asp301 and Lys302 of the bound HVDKDA sequence is mimicking the peptide bond of the physiological substrate of Csd1.

The interactions between the C-terminal tail residues of Csd2_121–308_ and Csd1_125–312_ are primarily mediated by the main-chain atoms between Asp301 and Asp303 of Csd2_121–308_, and Wat2 in the Zn^2+^-coordination sphere of Csd1_125–312_ (Fig 7B). The Asp301–Asp303 segment of the Csd2 tail covers the Csd1 active site; Wat2, one of the two Zn^2+^-coordinating water molecules in the Csd1 active site, makes three interactions with the Csd2 tail [Asp301 carbonyl oxygen (2.99 Å), Lys302 amide nitrogen (with 2.89 Å), and Asp303 amide nitrogen (with 2.97 Å)]. Both carbonyl oxygen and amide nitrogen atoms of Csd2 Val300 interact with Thr247 on Loop 3 of Csd1, while the carbonyl oxygen of Csd2 Asp303 interacts with Thr170 on Loop 1 of Csd1. The direction of the Csd2 peptide bound to Csd1 is consistent with the recent crystal structure of *Staphylococcus aureus* LytM in complex with tetraglycine phosphinate, a transition-state analog for hydrolysis of the penta-glycine bridge of the peptidoglycan cross-links [46]. This work helps to identify the substrate recognition and binding as well as describe details of the catalytic mechanism for cleaving the penta-glycine bridge by *S*. *aureus* LytM. The two Zn^2+^-coordinating water molecules of Csd1_125–312_ (chain C) superimpose nicely with two oxygen atoms of the phosphinate group in *S*. *aureus* LytM; they also superimpose well with two side-chain oxygen atoms of Glu74 from the α3 helix of Domain 1 in *H*. *pylori* Csd3 (Fig 7C). The oxyanion intermediate of the reaction catalyzed by *S*. *aureus* LytM was suggested to be stabilized by Tyr204 [46]. Sequence alignment indicates that this tyrosine residue is replaced by a histidine residue (His160) in *H*. *pylori* Csd1, as well as in Csd2 and Csd3 (His156 in Csd2 and His250 in Csd3) (Fig 7D). His160 of Csd1 is part of the disordered region in Loop 1 and is not included in the model. His156 of Csd2 is in proximity of the active site. However, His250 of Csd3 is located on Loop I, which protrudes from the LytM domain and is far from the active site.

## Discussion

Over the past years, many members of the M23 metallopeptidase family have been identified and biochemically characterized among the Gram-positive and Gram-negative bacteria. They have been implicated in a variety of important processes, including cell division, cell elongation, cell-shape determination, and sporulation [10,26,48–53]. Many of its members are recognized by the catalytic LytM domain, which possesses two characteristic motifs for Zn^2+^-binding (HxxxD and HxH) and a conserved histidine residue for catalysis. Some members of the M23 metallopeptidase family are expected to be non-peptidases, as their LytM domains have the degenerated active sites. There are many examples for inactive homologs of enzymes acquiring new functions as binding proteins [54]. The *E*. *coli* LytM proteins (EnvC and NlpD) are involved in the cell-division process by activating the septal peptidoglycan hydrolysis by amidase [55]. The LytM domain of EnvC lacks all five key residues and its structure showed that the catalytic metal ion is missing from the active site. Mutational analyses revealed that residues around the degenerated active site are critical for amidase activation *in vivo* and *in vitro* [56]. In *Bacillus subtilis*, the membrane protein SpoIIQ functions as a structural component by interacting with another membrane protein SpoIIIAH to form the core of a channel that connects the two compartments during sporulation [52,57,58]. SpoIIQ contains an extracellular LytM domain having the degenerated active site that misses one of three metal-binding residues (Ser119 instead of His of the HxxxD motif) and the proposed catalytic residue (Ser169 instead of His), resulting in the absence of a metal ion. The structure of the SpoIIQ and SpoIIIAH complex revealed that SpoIIIAH recognizes a region that protrudes from the N-terminus of the SpoIIQ LytM domain. Both *H*. *pylori* Csd1 and Csd2 belong to the M23 metallopeptidase family. However, they differ significantly in their LytM domains; all five key residues are conserved in Csd1, whereas only three of the five key residues are conserved in Csd2.

Here, we have identified the 1:1 complex formation between Csd1 (HP1543) and Csd2 (HP1544) by solving the crystal structure of the Csd1_125–312_-Csd2_121–308_ heterodimer and also by performing SEC-MALS in solution. This finding is in agreement with the previous genetic study that demonstrated that the deletion of either *csd1* or *csd2*, or deletion of both genes led to a similar increase in tetra-pentapeptide cross-linked dimers in muropeptide composition and impaired helical twist resulting in the curved-rod morphology [10]. Therefore, one can imagine that Csd1 and Csd2 might form a complex to function in *H*. *pylori* cells. Our structures show that the active site of the Csd1 LytM domain is bound with a Zn^2+^ ion but the Csd2 LytM domain is degenerate and no metal is bound. The present Csd1_125–312_-Csd2_121–308_ heterodimer is the first structure of the complex between two LytM homologs. The complex structure reveals that Csd1 and Csd2 make a heterodimer through their helical domains and dimerization does not affect the active site of the LytM domain. The helical domains of Csd1 and Csd2 are not conserved in other LytM proteins and show no structural similarity to other known protein structures. In both Csd1 and Csd2, the helical domain consists of a helix preceding the N-terminal side of the LytM domain and three helices following the C-terminal side of the LytM domain.

The *ccmA* gene of *H*. *pylori* is adjacent to *csd1* in the three-gene shape locus [10]. Deletion of the *ccmA* gene led to similar alterations in the muropeptide composition observed for single or double gene deletion for *csd1* and *csd2* [10]. CcmA lacks any recognizable peptidase motif but is a homolog of bactofilins, which are a widespread class of bacterial filament-forming proteins and serve as cytoskeletal scaffolds in various cellular pathways [59]. The highly polymerized CcmA protein may interact with Csd1 and Csd2 proteins to facilitate their localization in forming potential cellular machinery for precisely processing peptidoglycan. However, we could not test the interactions, because the CcmA protein formed high-molecular aggregates when it was overexpressed in *E*. *coli*, as one might anticipate from its inherent property to form filaments.

A recent study described the synthesis of a phosphinic acid-based inhibitor against Csd4 from *H*. *pylori* [60]. It demonstrated that the hydrophilic small-molecule inhibitor of Csd4 can cross the outer membrane of *H*. *pylori* and cause cell straightening, suggesting that Csd4 is a potential novel target for antibiotic development. Similarly, small-molecule inhibitors of Csd1 and Csd2 could be developed in the discovery of potential new antibiotics. Our work lays the foundation for such efforts.

## Supporting Information

S1 Fig Two structures of Csd1_125–312_-Csd2_121–308_ heterodimer. Two structures of Csd1_125–312_-Csd2_121–308_ heterodimer (Csd1-Csd2 dimer I and dimer II) as determined using two different data sets are shown in ribbon diagram. Both heterodimer structures contain two copies of the heterodimer in the asymmetric unit: an AB dimer, formed by Csd1_125–312_ (chain A, light blue) and Csd2_121–308_ (chain B, light green), and a CD dimer, formed by Csd1_125–312_ (chain C, colored in sky blue) and Csd2_121–308_ (chain D, yellow-green). A black dotted line divides two copies of the heterodimer in the asymmetric unit, which are related by non-crystallographic two-fold symmetry. Anomalous difference electron densities for Zn^2+^ ions in chains A and C of dimer II are shown in white-grey mesh (right). The omit mF_o_ − DF_c_ map for Zn^2+^ ions in all four chains of Csd1 are shown in yellow mesh (bottom). No metal ion is bound to Csd2 chains.
(TIF)

S2 Fig Three different types of Zn^2+^-coordination are observed in the Csd1 LytM domains. (A) Two copies of the Csd1_125–312_-Csd2_121–308_ heterodimer (AB dimer and CD dimer) in the structure of Csd1-Csd2 dimer I are superimposed and shown in ribbon diagram (left). Detailed views of the region covering the sequence of Leu145–Leu172 (colored in red) in (i) Csd1_125–312_ chain C of the Csd1-Csd2 dimer I (left), (ii) Csd1_125–312_ chain A of the Csd1-Csd2 dimer I (middle), and (iii) Csd1_125–312_ chain A in the Csd1-Csd2 dimer II (right) are shown in the black boxes. The region covering Leu145–Leu172 is structurally most divergent among different chains of Csd1. The dotted lines represent disordered loops. An extra α-helix (labeled as α1a) is formed in Csd1_125–312_ chain A in both structures of Csd1-Csd2 dimer I and dimer II. Four chains of Csd1_125–312_ have different lengths of Loop 1. Loop 1 in Csd1_125–312_ chain A of the Csd1-Csd2 dimer I (middle) is much shorter than those in chain C of the Csd1-Csd2 dimer I structure (left) and chain A of the Csd1-Csd2 dimer II structure (right). (B) Detailed views of three different types of Zn^2+^-coordination by the LytM domain of Csd1. The central β-sheet is shown in ribbon diagram, with Zn^2+^-coordinating residues (His164, His169 and Asp173 of the HxxxD motif, His250 and His252 of the HxH motif, and highly conserved His219) in stick models. Dotted lines represent direct Zn^2+^-coordination or close contacts. In the canonical coordination, the Zn^2+^ ion is coordinated by three conserved ligands (His169, Asp173, and His252) and two water molecules (Wat1 and Wat2). His164 is disordered in this model. In non-canonical coordination A, the Zn^2+^ ion is coordinated by Asp173, His219, His250, and His252. His169 is far away from the Zn^2+^ ion and is not included in this Fig Again, His164 is disordered in this model. In non-canonical coordination B, the Zn^2+^ ion is coordinated by His164 and two conserved ligands (Asp173 and His252) and a water molecule (Wat1). His169 is moved away from the Zn^2+^ ion. His164 replaces His169 in canonical coordination to act as the metal ligand.
(TIF)

S3 Fig Dimer interface within the Csd1_125–312_-Csd2_121–308_ heterodimer I. (A) The heterodimer interface between Csd1_125–312_ (chain C, shown in the electrostatic surface diagram) and Csd2_121–308_ (chain D, shown in ribbon diagram). A salt bridge (SB) and three hydrogen bonds (HB1–3) are indicated by dotted circles. The AB dimer of heterodimer I, and AB and CD dimers of heterodimer II have highly similar interfaces. (B) Detailed views of the salt bridge and the hydrogen bond (HB1) in (A) are indicated by black dotted lines in upper and lower panels, respectively. Csd1_125–312_ (chain C) is in sky blue, while Csd2_121–308_ (chain D) is in yellow-green, as in Fig 6. (C) The electrostatic surface diagram (left) represents the positively charged surface of α4 helix in Csd1_125–312_ (chain C) in the hetero-dimer interface. Csd2_121–308_ (chain D) is shown in ribbon diagram (yellow-green). In the right panel, detailed views of two hydrogen bonds (HB2 and HB3) are indicated by black dotted lines. (D) Superimposition of α3 helix in Csd2_121–308_ (darker green) onto α4 helix of Csd1_125–312_ (yellow-green). Glu282 and Asp292 on the Csd2_121–308_ α3 helix, which structurally correspond to Arg286 and Arg296 of Csd1_125–312_, respectively, are shown in stick models.
(TIF)

S4 Fig Cell shape-determinant proteins Csd1, Csd2, and Csd3 from *H*. *pylori*. (A) Sequence alignment of cell shape-determinant proteins Csd1, Csd2, and Csd3 from *H*. *pylori* 26695 strain. Sequences of Csd1 (HP_Csd1; SWISS-PROT accession code: O26068), Csd2 (HP_Csd2; O26069), and Csd3 (HP_Csd3; O25247) were aligned using Clustal Omega [61] and the alignment figure was drawn using ESPript (http://espript.ibcp.fr) [62]. The secondary structures are presented above the aligned sequences. Two large black boxes indicate the helical domains in Csd1 and Csd2. In Csd3, they correspond to part of Domain 2 and the C-terminal helical region that associates with Domain 2 [63]. LytM domains are found between these black boxes. Two small blue boxes indicate the conserved HxxxD and HxH motifs in the LytM domains, with blue triangles corresponding to the Zn^2+^-coordinating residues of Csd1 and Csd3. (B) Comparison of LytM domains in *H*. *pylori* Csd1 (chain C in heterodimer model I), Csd2 (in homodimer), and Csd3 [63]. Dotted grey lines indicate disordered loops in Csd1 and Csd3 LytM domains. 3_10_-Helices are colored in yellow. A Zn^2+^ ion is bound to the LytM domains of Csd1 and Csd3, whereas no Zn^2+^ ion is bound to the Csd2 LytM domain. (C) Domain organizations of Csd1, Csd2, and Csd3 proteins in *H*. *pylori* 26695 strain. Colored boxes indicate the structurally characterized regions.
(TIF)

S1 Table Structural similarity searches with the Csd2 LytM domain. (DOC)

S2 Table Structural similarity searches with the Csd1 LytM domain. (DOC)
