# Supplementary figures and images for: Structural Basis of the Heterodimer Formation between Cell Shape-Determining Proteins Csd1 and Csd2 from Helicobacter pylori
Source: PLoS One. 2016 Oct 6;11(10):e0164243. doi: 10.1371/journal.pone.0164243 (PMC5053510; doi:10.1371/journal.pone.0164243)

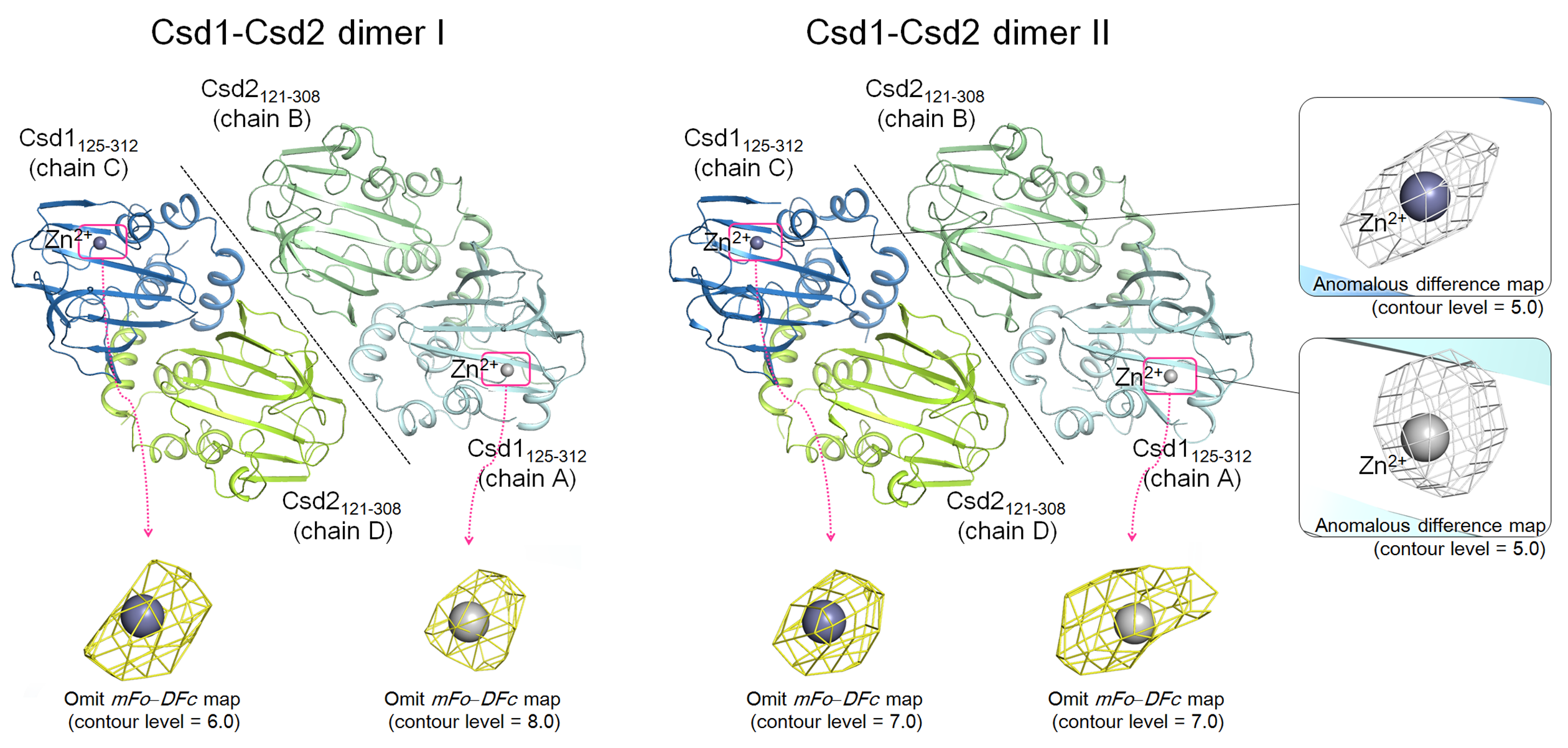

Supplement: S1 Fig — Two structures of Csd1125–312-Csd2121–308 heterodimer (Csd1-Csd2 dimer I and dimer II) as determined using two different data sets are shown in ribbon diagram. Both heterodimer structures contain two copies of the heterodimer in the asymmetric unit: an AB dimer, formed by Csd1125–312 (chain A, light blue) and Csd2121–308 (chain B, light green), and a CD dimer, formed by Csd1125–312 (chain C, colored in sky blue) and Csd2121–308 (chain D, yellow-green). A black dotted line divides two copies of the heterodimer in the asymmetric unit, which are related by non-crystallographic two-fold symmetry. Anomalous difference electron densities for Zn2+ ions in chains A and C of dimer II are shown in white-grey mesh (right). The omit mFo − DFc map for Zn2+ ions in all four chains of Csd1 are shown in yellow mesh (bottom). No metal ion is bound to Csd2 chains. (TIF) [file pone.0164243.s001.tif]

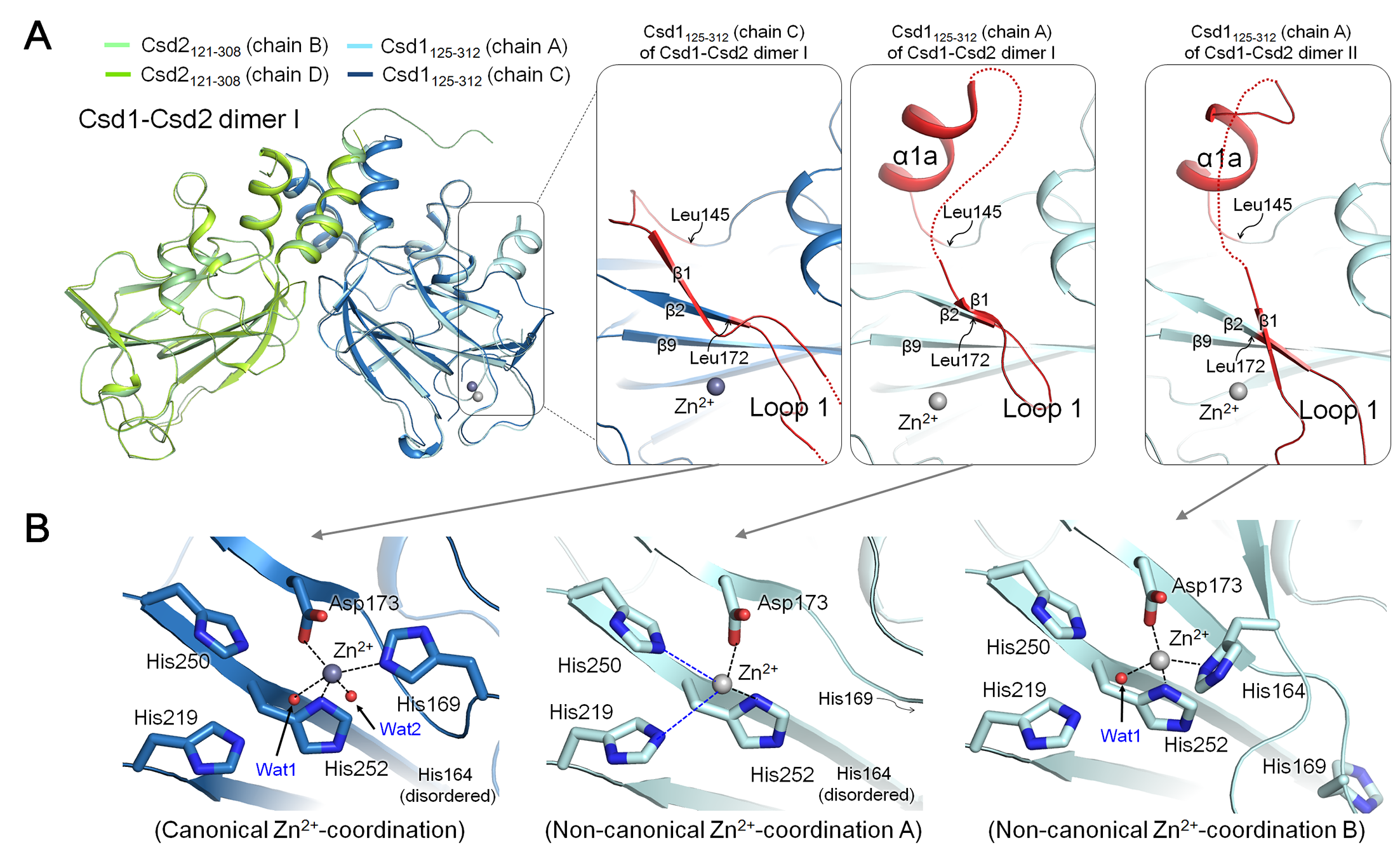

Supplement: S2 Fig — (A) Two copies of the Csd1125–312-Csd2121–308 heterodimer (AB dimer and CD dimer) in the structure of Csd1-Csd2 dimer I are superimposed and shown in ribbon diagram (left). Detailed views of the region covering the sequence of Leu145–Leu172 (colored in red) in (i) Csd1125–312 chain C of the Csd1-Csd2 dimer I (left), (ii) Csd1125–312 chain A of the Csd1-Csd2 dimer I (middle), and (iii) Csd1125–312 chain A in the Csd1-Csd2 dimer II (right) are shown in the black boxes. The region covering Leu145–Leu172 is structurally most divergent among different chains of Csd1. The dotted lines represent disordered loops. An extra α-helix (labeled as α1a) is formed in Csd1125–312 chain A in both structures of Csd1-Csd2 dimer I and dimer II. Four chains of Csd1125–312 have different lengths of Loop 1. Loop 1 in Csd1125–312 chain A of the Csd1-Csd2 dimer I (middle) is much shorter than those in chain C of the Csd1-Csd2 dimer I structure (left) and chain A of the Csd1-Csd2 dimer II structure (right). (B) Detailed views of three different types of Zn2+-coordination by the LytM domain of Csd1. The central β-sheet is shown in ribbon diagram, with Zn2+-coordinating residues (His164, His169 and Asp173 of the HxxxD motif, His250 and His252 of the HxH motif, and highly conserved His219) in stick models. Dotted lines represent direct Zn2+-coordination or close contacts. In the canonical coordination, the Zn2+ ion is coordinated by three conserved ligands (His169, Asp173, and His252) and two water molecules (Wat1 and Wat2). His164 is disordered in this model. In non-canonical coordination A, the Zn2+ ion is coordinated by Asp173, His219, His250, and His252. His169 is far away from the Zn2+ ion and is not included in this Fig Again, His164 is disordered in this model. In non-canonical coordination B, the Zn2+ ion is coordinated by His164 and two conserved ligands (Asp173 and His252) and a water molecule (Wat1). His169 is moved away from the Zn2+ ion. His164 replaces His169 in canonical coordina [file pone.0164243.s002.tif]

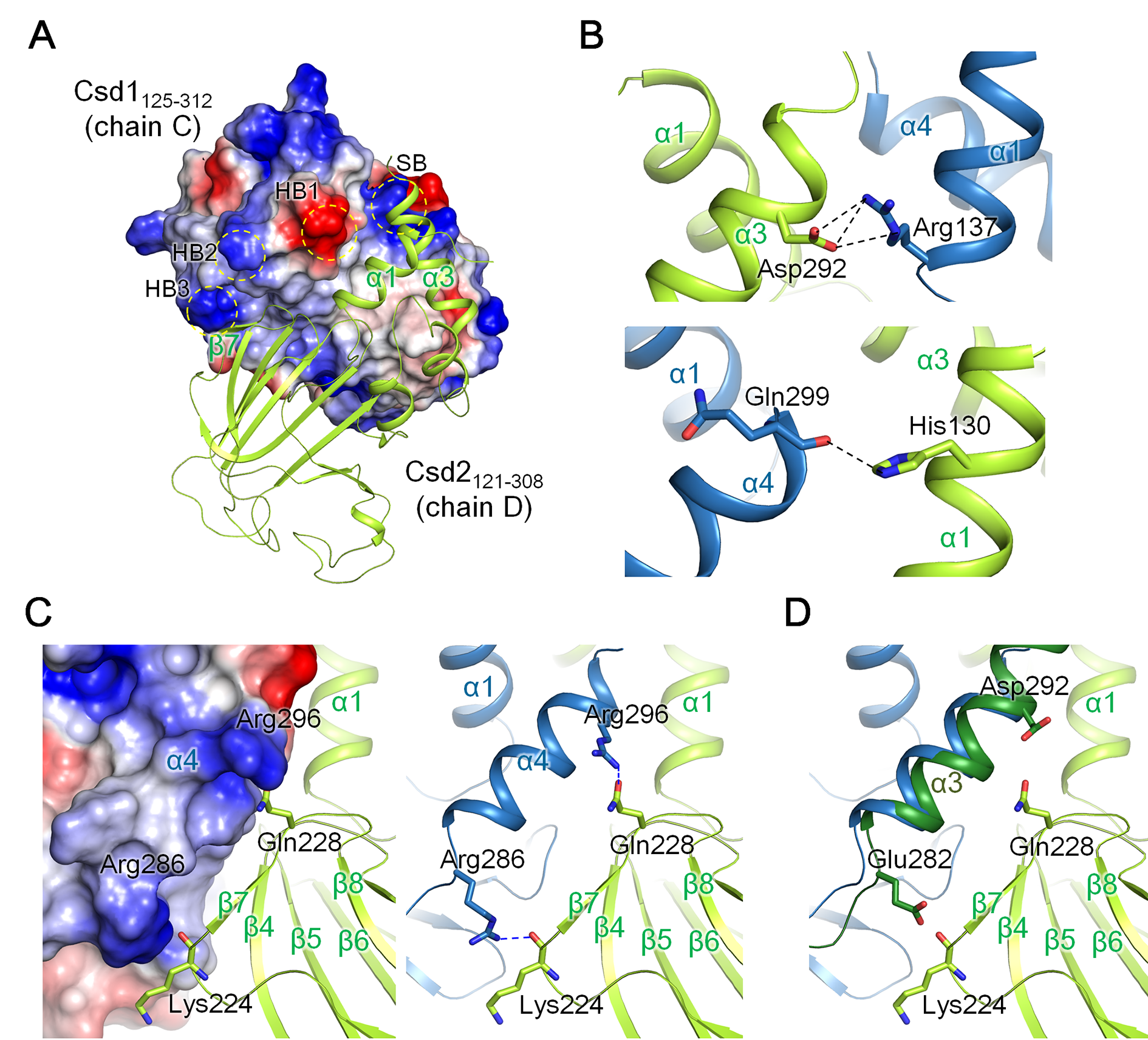

Supplement: S3 Fig — (A) The heterodimer interface between Csd1125–312 (chain C, shown in the electrostatic surface diagram) and Csd2121–308 (chain D, shown in ribbon diagram). A salt bridge (SB) and three hydrogen bonds (HB1–3) are indicated by dotted circles. The AB dimer of heterodimer I, and AB and CD dimers of heterodimer II have highly similar interfaces. (B) Detailed views of the salt bridge and the hydrogen bond (HB1) in (A) are indicated by black dotted lines in upper and lower panels, respectively. Csd1125–312 (chain C) is in sky blue, while Csd2121–308 (chain D) is in yellow-green, as in Fig 6. (C) The electrostatic surface diagram (left) represents the positively charged surface of α4 helix in Csd1125–312 (chain C) in the hetero-dimer interface. Csd2121–308 (chain D) is shown in ribbon diagram (yellow-green). In the right panel, detailed views of two hydrogen bonds (HB2 and HB3) are indicated by black dotted lines. (D) Superimposition of α3 helix in Csd2121–308 (darker green) onto α4 helix of Csd1125–312 (yellow-green). Glu282 and Asp292 on the Csd2121–308 α3 helix, which structurally correspond to Arg286 and Arg296 of Csd1125–312, respectively, are shown in stick models. (TIF) [file pone.0164243.s003.tif]

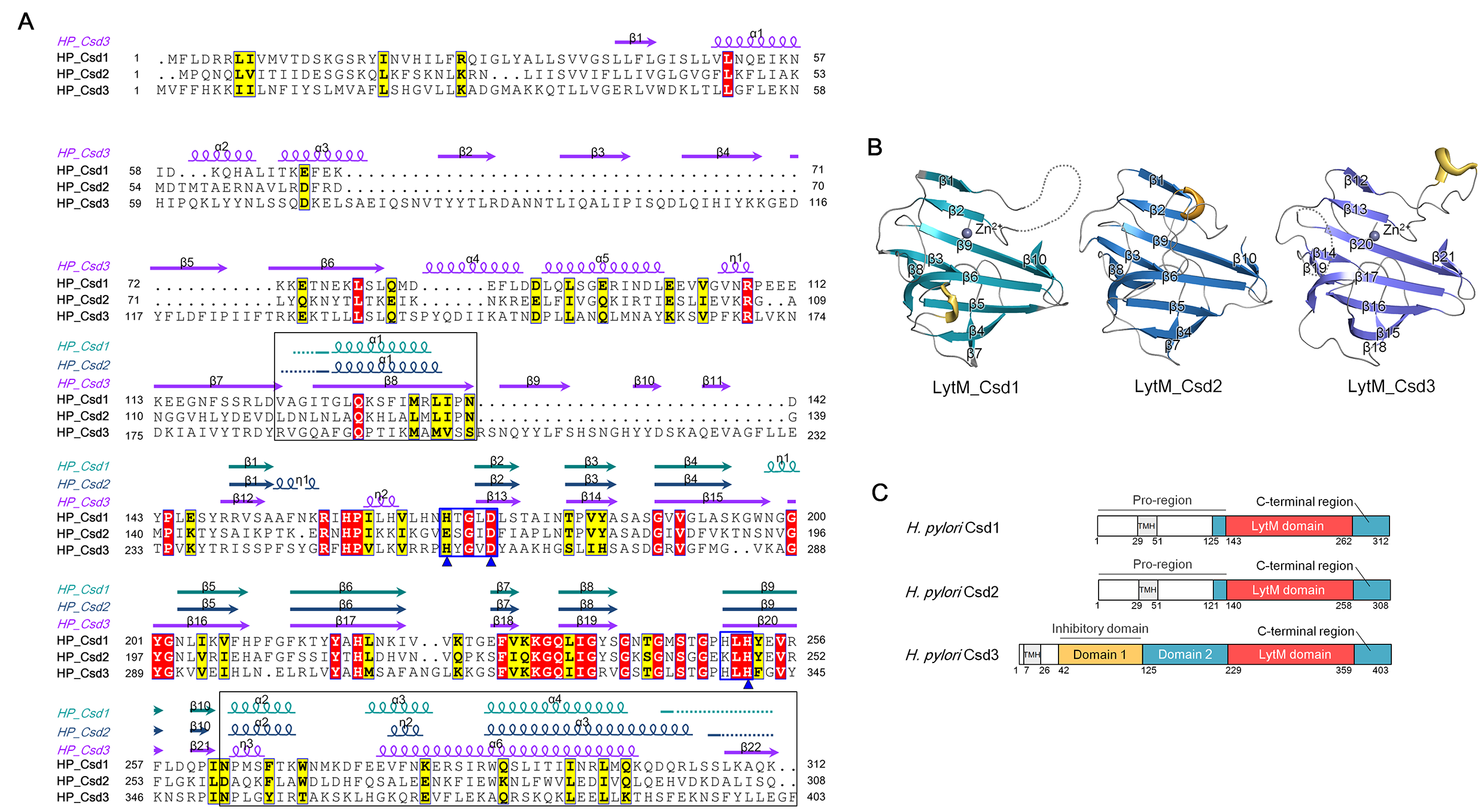

Supplement: S4 Fig — (A) Sequence alignment of cell shape-determinant proteins Csd1, Csd2, and Csd3 from H. pylori 26695 strain. Sequences of Csd1 (HP_Csd1; SWISS-PROT accession code: O26068), Csd2 (HP_Csd2; O26069), and Csd3 (HP_Csd3; O25247) were aligned using Clustal Omega [61] and the alignment figure was drawn using ESPript (http://espript.ibcp.fr) [62]. The secondary structures are presented above the aligned sequences. Two large black boxes indicate the helical domains in Csd1 and Csd2. In Csd3, they correspond to part of Domain 2 and the C-terminal helical region that associates with Domain 2 [63]. LytM domains are found between these black boxes. Two small blue boxes indicate the conserved HxxxD and HxH motifs in the LytM domains, with blue triangles corresponding to the Zn2+-coordinating residues of Csd1 and Csd3. (B) Comparison of LytM domains in H. pylori Csd1 (chain C in heterodimer model I), Csd2 (in homodimer), and Csd3 [63]. Dotted grey lines indicate disordered loops in Csd1 and Csd3 LytM domains. 310-Helices are colored in yellow. A Zn2+ ion is bound to the LytM domains of Csd1 and Csd3, whereas no Zn2+ ion is bound to the Csd2 LytM domain. (C) Domain organizations of Csd1, Csd2, and Csd3 proteins in H. pylori 26695 strain. Colored boxes indicate the structurally characterized regions. (TIF) [file pone.0164243.s004.tif]
