# Supplementary material for: Structural Basis of the Heterodimer Formation between Cell Shape-Determining Proteins Csd1 and Csd2 from Helicobacter pylori
Source: PLoS One. 2016 Oct 6;11(10):e0164243. doi: 10.1371/journal.pone.0164243 (PMC5053510; doi:10.1371/journal.pone.0164243)
Supplement: S1 Table — (DOC) [file pone.0164243.s005.doc]

**Table S1. Structural similarity searches with the Csd2 LytM domain.**

|  | Protein name | | PDB code | R.m.s. deviation | Z-score | Sequence identity |
| --- | --- | --- | --- | --- | --- | --- |
| Csd2  LytM domain | Three-domain protein | Lysostaphin peptidase (VC0503)  from *Vibrio cholera* | 2GU1 | 1.7 Å  (107 Cα) | 16.7 | 32% |
|  |  | Outer-membrane protein (NMB0315)  from *Neisseria meningitidis* | 3SLU | 1.9 Å  (106 Cα) | 16.0 | 39% |
|  |  | Cell shape determinant 3 (Csd3; HP0506)  from *Helicobacter pylori* | 4RNY | 1.8 Å  (102 Cα) | 15.5 | 28% |
|  | Pro-protein | Glycylglycine endopeptidase (LtyM)  from *Staphylococcus aureus* | 2B0P | 2.2 Å  (110 Cα) | 15.6 | 28% |
|  |  | Lysostaphin  from *Staphylococcus simulans* | 4QP5 | 2.0 Å  (111 Cα) | 15.7 | 26% |
|  | Non-peptidase protein | Murein hydrolase activator (EnvC)  from *Escherichia coli* | 4BH5 | 1.8 Å  (107 Cα) | 16.1 | 31% |
|  |  | Stage II sporulation protein Q (SpollQ)  from *Bacillus subtilis* | 3UZ0 | 2.0 Å  (113 Cα) | 14.6 | 31% |
