# Supplementary material for: Structural Basis of the Heterodimer Formation between Cell Shape-Determining Proteins Csd1 and Csd2 from Helicobacter pylori
Source: PLoS One. 2016 Oct 6;11(10):e0164243. doi: 10.1371/journal.pone.0164243 (PMC5053510; doi:10.1371/journal.pone.0164243)
Supplement: S2 Table — (DOC) [file pone.0164243.s006.doc]

**Table S2. Structural similarity searches with the Csd1 LytM domain.**

|  | Protein name | | PDB code | R.m.s. deviation | Z-score | Sequence identity |
| --- | --- | --- | --- | --- | --- | --- |
| Csd1  LytM domain | Three-domain protein | Lysostaphin peptidase (VC0503)  from *Vibrio cholera* | 2GU1 | 1.2 Å  (106 Cα) | 18.5 | 43% |
|  |  | Outer-membrane protein (NMB0315)  from *Neisseria meningitidis* | 3SLU | 1.3 Å  (104 Cα) | 18.2 | 46% |
|  |  | Cell shape determinant 3 (Csd3; HP0506)  from *Helicobacter pylori* | 4RNY | 1.0 Å  (100 Cα) | 17.5 | 36% |
|  | Pro-protein | Glycylglycine endopeptidase (LtyM)  from *Staphylococcus aureus* | 2B13 | 1.5 Å  (106 Cα) | 17.7 | 32% |
|  |  | Lysostaphin  from *Staphylococcus simulans* | 4QPB | 1.5 Å  (105 Cα) | 17.2 | 39% |
|  | Non-peptidase protein | Murein hydrolase activator (EnvC)  from *Escherichia coli* | 4BH5 | 1.2 Å  (104 Cα) | 17.8 | 29% |
|  |  | Stage II sporulation protein Q (SpollQ)  from *Bacillus subtilis* | 3UZ0 | 1.5 Å  (106 Cα) | 15.6 | 34% |
